# Long-Term Impact of Using Mobile Phones and Playing Computer Games on the Brain Structure and the Risk of Neurodegenerative Diseases: Large Population-Based Study

**DOI:** 10.2196/59663

**Published:** 2025-01-28

**Authors:** Yi Xiao, Sirui Zhang, Yuanzheng Ma, Shichan Wang, Chunyu Li, Yan Liang, Huifang Shang

**Affiliations:** 1 Department of Neurology West China Hospital, Sichuan University Chengdu China; 2 Laboratory of Neurodegenerative Disorders National Clinical Research Center for Geriatrics West China Hospital, Sichuan University Chengdu China

**Keywords:** electronic device, parkinsonism, dementia, aging, brain MRI, magnetic resonance imaging

## Abstract

**Background:**

Despite the increasing popularity of electronic devices, the longitudinal effects of daily prolonged electronic device usage on brain health and the aging process remain unclear.

**Objective:**

The aim of this study was to investigate the impact of the daily use of mobile phones/computers on the brain structure and the risk of neurodegenerative diseases.

**Methods:**

We used data from the UK Biobank, a longitudinal population-based cohort study, to analyze the impact of mobile phone use duration, weekly usage time, and playing computer games on the future brain structure and the future risk of various neurodegenerative diseases, including all-cause dementia (ACD), Alzheimer disease (AD), vascular dementia (VD), all-cause parkinsonism (ACP), and Parkinson disease (PD). All the characteristics of using mobile phones and playing computer games were collected through face-to-face interviews at baseline, and outcomes were extracted from the algorithmic combinations of self-reported medical conditions, hospital admissions, and death registries. In addition, a group of participants underwent magnetic resonance imaging (MRI) at follow-up. Cox regression and linear regression were performed.

**Results:**

The study included over 270,000 participants for risk analysis, with a mean baseline age of approximately 55.85 (SD 8.07) years. The average follow-up duration was approximately 13.9 (SD 1.99) years. Lengthy mobile phone use was associated with a reduced risk of ACD (2-4 years: hazard ratio [HR] 0.815, 95% CI 0.729-0.912, *P*<.001; 5-8 years: HR 0.749, 95% CI 0.677-0.829, *P*<.001; >8 years: HR 0.830, 95% CI 0.751-0.918, *P*<.001), AD (5-8 years: HR 0.787, 95% CI 0.672-0.922, *P*=.003), and VD (2-4 years: HR 0.616, 95% CI 0.477-0.794, *P*<.001; 5-8 years: HR 0.729, 95% CI 0.589-0.902, *P*=.004; >8 years: HR 0.750, 95% CI 0.605-0.930, *P*=.009) compared to rarely using mobile phones. Additionally, lengthy mobile phone use was linked to a decreased risk of ACP (5-8 years: HR 0.747, 95% CI 0.637-0.875, *P*<.001; >8 years: HR 0.774, 95% CI 0.663-0.904, *P*=.001) and PD (5-8 years: HR 0.760, 95% CI 0.644-0.897, *P*=.001; >8 years: HR 0.777, 95% CI 0.660-0.913, *P*=.002) in participants older than 60 years. However, higher weekly usage time did not confer additional risk reduction compared to lower weekly usage of mobile phones. The neuroimaging analysis involved 35,643 participants, with an average duration of approximately 9.0 years between baseline and neuroimaging scans. Lengthy mobile phone use was related to a thicker cortex in different areas of the brain.

**Conclusions:**

Lengthy mobile phone use is associated with a reduced risk of neurodegenerative diseases and improved brain structure compared to minimal usage. Our research provides valuable background knowledge for future studies on the impact of modern electronic devices on brain health.

## Introduction

Aging and digitalization are two key issues of the current era. The prevalence of neurodegenerative diseases is projected to increase with the aging of the population in the future [1]. Dementia and parkinsonism rank as the top two most prevalent neurodegenerative diseases, with Parkinson disease (PD) being the fastest growing among them [2]. Since disease-modifying treatments are still under research, early detection and prevention of neurodegenerative disease risk factors are crucial for reducing the disease burden on the health care system worldwide.

As digital technologies develop, the use of electronic devices is becoming increasingly popular, raising concerns about their impact on health. However, previous studies have reported controversial results. Electronic devices produce low-intensity electromagnetic fields and microwave radiation during operation, with mobile phones being particularly relevant due to their proximity to the head during use. Hypotheses have been proposed suggesting that low-intensity electromagnetic fields are associated with an increased risk and younger onset age of neurodegenerative diseases [3-5]. Conversely, some argue that exposure to low-intensity electromagnetic fields reduces the risk of Alzheimer disease (AD) [6]. Studies examining the association between mobile phone use and dementia risk have reported conflicting findings, with some suggesting a decreased risk, while others propose no association or even an increased risk [7,8]. Animal and in vivo studies have also yielded conflicting results, further complicating the understanding of the relationship between electronic device use and neurodegenerative diseases [9-11]. Studies on PD specifically have provided mixed evidence, with 1 study reporting no association, while another suggested a potential protective effect in males [8,12].

Overall, controversial hypotheses have been put forward by different studies, and there is a lack of convincing real-world evidence on this topic, especially in neuroepidemiology research. Furthermore, although changes in the brain structure and activity related to excessive phone use have been widely studied, less attention has been paid to the effects of daily electronic use [13,14]. For individuals who are not addicted to electronic devices, the impact of daily electronic use on the brain remains unclear. Compared to previous studies, this research further explored the details of the habit of electronic device use and its relationship with brain aging in participants of different ages.

To address the aforementioned research gaps, our study was designed to meet the following objectives: (1) the relationship between baseline usage of mobile phone/computers and the risk of developing neurodegenerative diseases and (2) the relationship between baseline daily usage of mobile phones/computers and the subsequent individual brain structure to explore the association between mobile phone/computer usage and brain aging. Clinical and imaging data were sourced from the UK Biobank. The findings of our study will offer valuable insights into the influence of electronic device usage on neurodegenerative processes.

## Methods

### Participants

The UK Biobank is a comprehensive longitudinal population-based cohort that enrolled 502,376 participants from 2006 to 2010 in the United Kingdom. In this study, we included participants who had baseline electronic device usage data and covariate data. The exclusion criteria were a history of the following diseases at baseline: dementia, stroke, PD, multiple sclerosis, demyelinating disorders, and other neurodegenerative diseases.

### Ethical Considerations

The UK Biobank was approved (number 21/NW/0157) by the North West Multi-centre Research Ethics Committee (MREC). Consent were acquired from participants at the recruitment center for the study. Compensation and consent details are available on the UK Biobank website [15]. This manuscript was approved for publication by the UK Biobank, and data in the UK Biobank were accessible for health-related research with the approval of the UK Biobank Ethics Advisory Committee. All the data used in the study were anonymized, and no identifiable features were included.

### Electronic Device Use

Data on the use of electronic devices were extracted from the UK Biobank database [16] and transferred into category variables: plays computer games (3 levels: never/rarely, sometimes, often) and length of mobile phone use (5 levels: never used a mobile phone at least once per week, 1 year or less, 2-4 years; 5-8 years, >8 years). For those who used mobile phones, the following data were extracted: weekly usage of mobile phones in the past 3 months (2 levels: <5 minutes, ≥5 minutes), hands-free device/speakerphone use with mobile phones in the past 3 months (2 levels: never/almost never, used), differences in mobile phone use compared to the previous 2 years (3 levels: no change, more frequent, less frequent), and usual side of the head for mobile phone use (3 levels: left, right, equally left and right). For details of the answer to each question and their form in the models, see Multimedia Appendix 1.

### Neuroimaging

The details of the magnetic resonance imaging (MRI) performed can be found on the UK Biobank website and in previous studies [17-19]. In brief, participants were invited to attend the research centers for follow-up and further neuroimaging assessment from 2014. Imaging scans were conducted in 3 imaging centers with identical scanners and fixed platforms to minimize heterogeneity. Those who had a neurological disease before the imaging scan were excluded from the analysis.

Brain imaging was acquired with 3T Siemens Skyra (software platform VD13) using a 32-channel receive head coil. T1 images were collected at 1 mm isotropic resolution using 3D MPRAGE acquisition, with the superior-inferior field of view being 256 mm. Several structural metrics were provided by the UK Biobank using Freesurfer. The most segmented brain imaging metrics were selected to obtain the most detailed changes. We used metrics processed by Destrieux (a2009s) parcellation in Freesurfer to assess the area, mean thickness, and volume of the brain in 148 areas [20].

Diffusion tensor imaging (DTI) is an advanced MRI technology that can detect microstructural abnormalities in white matter (WM) before the visual injury emerges. Diffusion data were collected with 2 b-values (1000 and 2000 s/mm^2^) at 2 mm spatial resolution, with a multiband acceleration factor of 3. Fifty distinct diffusion-encoding directions were acquired for each diffusion-weighted shell. The diffusion preparation was a standard (“monopolar”) Stejskal-Tanner pulse sequence. We used 5 DTI metrics provided by the UK Biobank: fractional anisotropy (FA), mean diffusion (MD), orientation dispersion (OD), intracellular volume fraction (ICVF), and isotropic (free) water volume fraction (ISOVF) [17]. FA and MD were calculated by fitting a voxel-wise diffusion tensor model through DTIFIT in FSL. The OD, ICVF, and ISOVF were calculated by fitting neurite orientation dispersion and density imaging models using accelerated microstructure imaging via convex optimization [21,22]. Low FA or high MD indicate a higher overall deficit in WM fiber integrity. Specifically, OD represents the overall coherence of fibers, the ICVF represents axonal/neurite density, and the ISOVF indicates the free-water fraction [22].

### Diagnosis

We used the diagnosis of neurodegenerative diseases (dementia outcomes and parkinsonism outcomes) from category 47 and category 50: all-cause dementia (ACD), AD, vascular dementia (VD), all-cause parkinsonism (ACP), and PD. These outcomes were defined through algorithmic combinations of self-reported medical conditions, hospital admissions, and death registries [23].

### Statistical Analysis

Data were described as mean (SD) values for continuous variables and counts (percentages) for category variables. Continuous variables were compared using the *t* test, and category variables were compared using the chi-square test or the Fisher exact test. We used Cox regression analysis to evaluate the risk of dementia. Participants were considered at risk for dementia or parkinsonism from baseline (2006-2010) and were followed up to the date of the first diagnosis, death, loss of follow-up, or the last hospital admission.

The covariates included age, sex, the BMI, smoking (ever or never), drinking (ever or never), hypertension, diabetes, education (college/university degree or lower degree), the polygenic risk score, socioeconomic status (Townsend deprivation index, a score calculated according to the output area in which participants’ postcodes were located and the preceding national census output areas), the average total household income before tax, the mental health score, and physical activity. Using scaled Schoenfeld residuals to test proportional hazards, there was no indication of a violation of the hypothesis. In the sensitivity analysis, we further conducted several models: (1) model 1 excluded those diagnosed with outcome diseases within 5 years from baseline to exclude the potential reverse causality, (2) model 2 only included diagnoses that were from the hospital or death registration; (3) model 3 was used to replace the polygenic risk score (PRS) with the status of the *apolipoprotein ε4* gene, and (4) model 4 divided patients into 2 groups according to age (60 years old) at baseline to exclude the influence of age.

Linear regression models were used with variables using electronic devices as independent variables and neuroimaging metrics as dependent variables. Sex, age, ethnicity, the BMI, smoking, drinking, hypertension, diabetes, education, socioeconomic status, the WM hyperintensity volume, the whole WM volume, handedness (only for the usual use of the side of the mobile phone), and the average total household income before tax were included as covariates [24]. In the sensitivity analysis, we further adjusted the mental health score and physical activity. The variance inflation factor was tested and controlled under 5 to avoid multicollinearity. We used Bonferroni-corrected *P* values to determine the statistical significance: .05/5 for disease risk analysis, .05/(48 tracts×5 metrics) for DTI, and .05/148 regions for the surface, thickness, and volume in linear regression models. All analyses were performed using R version 4.1.2 (R Foundation for Statistical Computing).

## Results

### Demographic Characteristics of Participants Included in Risk Analysis

**Figure 1:**
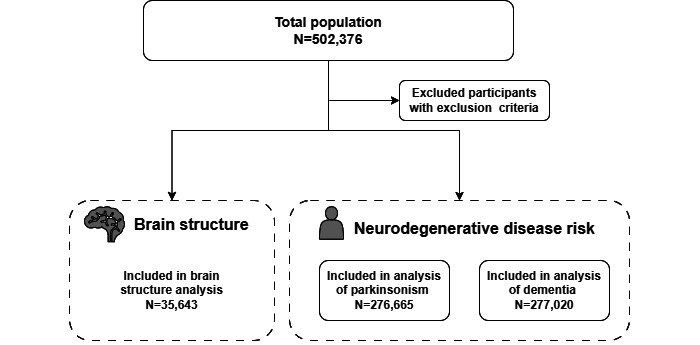
Inclusion flowchart.

The inclusion flowchart is displayed in Figure 1 (detailed version in Multimedia Appendix 1), and the demographic characteristics of the participants are shown in Table 1. A total of 277,020 and 276,665 participants were included in the Cox regression model for dementia and parkinsonism, respectively. The mean age of the dementia and parkinsonism analysis groups at baseline was 55.85 (SD 8.07) years and 55.86 (SD 8.07) years, respectively. Half of the included participants (dementia group: n=139,166, 50%; parkinsonism group: n=139,276, 50%) were female. The mean duration of follow-up was 13.90 (SD 1.99) years and 13.89 (SD 2.01) years for the dementia and parkinsonism groups, respectively. We further compared the characteristics of the included and excluded participants (Tables S1 and S2 in Multimedia Appendix 1). The participants in the included risk analysis were younger, had higher education, a lower Townsend deprivation index, and a higher income compared to those excluded from the analysis.

**Table 1:** Demographic characteristics of the included population (N=502,376).

Variables		Parkinsonism group (n=276,665)	Dementia group (n=277,020)
**Usual side of the head for mobile phone use, n (%)**			
	Left	84,079 (30)	84,181 (30)
	Right	139,275 (50)	139,429 (50)
	Equally left and right	15,119 (6)	15,131 (6)
	Do not need to answer	38,192 (14)	38,279 (14)
**Weekly usage of mobile phones in the past 3 months, n (%)**			
	<5 minutes	46,861 (17)	46,939 (17)
	≥5 minutes	191,612 (69)	191,802 (69)
	Do not need to answer	38,192 (14)	38,279 (14)
**Difference in mobile phone use compared to the previous 2 years, n (%)**			
	No change	124,344 (45)	124,496 (45)
	More frequent	31,551 (11)	31,600 (11)
	Less frequent	82,578 (30)	82,645 (30)
	Do not need to answer	38,192 (14)	38,279 (14)
**Plays computer games, n (%)**			
	Never/rarely	218,586 (79)	218,873 (79)
	Sometimes	48,861 (18)	48,915 (18)
	Often	9218 (3)	9232 (3)
**Hands-free device/speakerphone use with mobile phones in the past 3 months, n (%)**			
	Never/almost never	191,825 (69)	192,057 (69)
	Used	46,648 (17)	46,684 (17)
	Do not need to answer	38,192 (14)	38,279 (14)
**Length of mobile phone use, n (%)**			
	Never used a mobile phone at least once per week	38,192 (14)	38,279 (14)
	≤1 year	6316 (2)	6325 (2)
	2-4 years	44,446 (16)	44,507 (16)
	5-8 years	85,160 (31)	85,263 (31)
	>8 years	102,551 (37)	102,646 (37)
Age (years), mean (SD)		55.85 (8.07)	55.86 (8.07)
**Sex, n (%)**			
	Female	139,166 (50)	139,276 (50)
	Male	137,499 (50)	137,744 (50)
BMI, mean (SD)		27.29 (4.65)	27.29 (4.65)
**Smoking, n (%)**			
	Yes	151,799 (55)	152,022 (55)
	No	124,866 (45)	124,998 (45)
**Drinking, n (%)**			
	Yes	9187 (3)	9198 (3)
	No	267,478 (97)	267,822 (97)
**Hypertension, n (%)**			
	Yes	69,764 (25)	69,863 (25)
	No	206,901 (75)	207,157 (75)
**Diabetes, n (%)**			
	Yes	264,018 (95)	264,358 (95)
	No	12,647 (5)	12,662 (5)
**Education, n (%)**			
	College or higher	172,135 (62)	172,364 (62)
	Lower than college	104,530 (38)	104,656 (38)
AD^a^ polygenic risk score, mean (SD)		–0.14 (1.02)	0.05 (0.99)
Townsend deprivation index, mean (SD)		–1.50 (2.97)	–1.50 (2.97)
**Average total household income before tax (US $), n (%)**			
	22,862-39,372	53,376 (19)	53,485 (19)
	39,373-66,044	67,464 (24)	67,579 (24)
	66,045-127,010	74,930 (27)	75,011 (27)
	Do not know	62,972 (23)	63,009 (23)
	>127,010	17,923 (6)	17,936 (6)
Mental health score, mean (SD)		4.31 (3.25)	4.31 (3.25)
**Physical activity, n (%)**			
	Not meeting the criteria	41,256 (15)	41,183 (15)
	Meeting the criteria	235,764 (85)	235,482 (85)

^a^AD: Alzheimer disease.

### Relationship Between Electronic Device Use and Dementia

In the follow-up, we included 3007 (1.1%) participants with ACD, 1231 (0.4%) with AD, and 631 (0.2%) with VD. Lengthy mobile phone use (≥2 years) at baseline was related to a lower risk of ACD and VD compared to never using a mobile phone at least once per week (Table 2 and Table S3 in Multimedia Appendix 1). Additionally, participants who had used mobile phones for 5-8 years had a significantly lower risk of AD.

**Table 2:** Association between the length of mobile phone use and the risk of dementia.

Outcome and length of mobile phone use (years)		HR^a^ (95% CI)	Outcomes, n (%)	Participants, n (%)	*Z* value	*P* value
**ACD^b^**						
	Reference^c^	1.000 (1.000-1.000)	793 (2.1)	38,279 (14)	1.000	N/A^d^
	≤1	1.002 (0.824-1.219)	115 (1.8)	6325 (2)	0.022	.98
	2-4	0.815 (0.729-0.912)	510 (1.2)	44,507 (16)	–3.566	<.001^e^
	5-8	0.749 (0.677-0.829)	750 (0.9)	85,263 (31)	–5.600	<.001^e^
	>8	0.830 (0.751-0.918)	839 (0.8)	102,646 (37)	–3.630	<.001^e^
**AD^f^**						
	Reference^c^	1.000 (1.000-1.000)	321 (0.8)	38,279 (14)	1.000	N/A
	≤1	1.151 (0.860-1.540)	53 (0.8)	6325 (2)	0.945	.35
	2-4	0.919 (0.774-1.091)	229 (0.5)	44,507 (16)	–0.968	.33
	5-8	0.787 (0.672-0.922)	311 (0.4)	85,263 (31)	–2.973	.003^e^
	>8	0.835 (0.711-0.979)	317 (0.3)	102,646 (37)	–2.215	.03
**VD^g^**						
	Reference^c^	1.000 (1.000-1.000)	186 (0.5)	38,279 (14)	1.000	N/A
	≤1	0.832 (0.539-1.284)	23 (0.4)	6325 (2)	–0.830	.41
	2-4	0.616 (0.477-0.794)	89 (0.2)	44,507 (16)	–3.738	<.001^e^
	5-8	0.729 (0.589-0.902)	163 (0.2)	85,263 (31)	–2.910	.004^e^
	>8	0.750 (0.605-0.930)	170 (0.2)	102,646 (37)	–2.619	.009^e^

^a^HR: hazard ratio.

^b^ACD: all-cause dementia.

^c^Never used a mobile phone at least once per week.

^d^N/A: not applicable.

^e^Significant at *P*<.05/5.

^f^AD: Alzheimer disease.

^g^VD: vascular dementia.

To exclude the influence of reverse causality, several sensitivity analyses were conducted. After excluding participants who developed outcomes within 5 years after baseline, we found that lengthy mobile phone use was related to a lower risk of ACD (mobile phone use≥2 years), VD (mobile phone use=2-4 years), and AD (mobile phone use=5-8 years), as shown in Table S4 in Multimedia Appendix 1. After excluding participants whose source of outcomes was not from the hospital or death registration, we found that lengthy mobile phone use (≥2 years) was related to a lower risk of ACD and VD (Table S5 in Multimedia Appendix 1). To analyze the influence of *apolipoprotein ε4*, we adjusted the status of *apolipoprotein ε4* instead of the PRS and found that lengthy mobile phone use was related to a decreased risk of ACD (mobile phone use≥2 years) and VD (mobile phone use=2-4 years), as shown in Table S6 in Multimedia Appendix 1.

To exclude the influence of age, we conducted a separate analysis based on participants’ age at baseline. We found that lengthy mobile phone use was related to a decreased risk of ACD (mobile phone use≥2 years) and AD (mobile phone use≥5 years) in participants younger than 60 years (Table S7 in Multimedia Appendix 1). Lengthy mobile phone use was also related to a decreased risk of ACD (mobile phone use≥2 years), AD (mobile phone use≥5 years), and VD (mobile phone use≥2 years) in participants older than 60 years (Table S8 in Multimedia Appendix 1).

### Relationship Between Electronic Device Use and Parkinsonism

In the follow-up, we included 1578 (0.6%) participants with ACP and 1415 (0.5%) with PD. Often playing computer games was related to a lower risk of ACP (hazard ratio [HR] 0.604, 95% CI 0.425-0.859; *P*=.005) and PD (HR 0.574, 95% CI 0.392-0.841; *P*=.004) compared to never or rarely playing computer games (Table S9 in Multimedia Appendix 1). However, after excluding those who developed outcomes within 5 years, none of the above results remained statistically significant (Table S10 in Multimedia Appendix 1). We excluded participants whose source of outcomes was not from the hospital or death registration and reached a similar result that often playing computer games was related to a lower risk of ACP and PD (Table S11 in Multimedia Appendix 1). After age stratification, in participants younger than 60 years, we found that the association between often playing computer games and a lower risk of ACP did not reach statistical significance (HR 0.393, 95% CI 0.175-0.844; *P*=.02; Table S12 in Multimedia Appendix 1). However, lengthy mobile phone use (≥5 years) was related to a decreased risk of ACP and PD in participants older than 60 years (Table S13 in Multimedia Appendix 1).

### Demographic Characteristics of Participants Included in Neuroimaging Analysis

The demographic characteristics of participants who underwent neuroimaging analysis are displayed in Table S14 in Multimedia Appendix 1. A total of 35,643 participants were included, 48% (n=17,109) of them were male, and the mean age at baseline was 54.77 (SD 7.51) years. The mean duration between baseline and the neuroimaging scan was 8.97 (SD 1.73) years.

### Relationship Between Electronic Device Use and the Brain Structure

Areas where the brain structure metrics at follow-up were related to the use of electronic devices at baseline are displayed in Tables 3 and 4. Using mobile phones for a long duration (≥2 years) was related to a thicker cortex in the left superior temporal sulcus. In addition, lengthy mobile phone use (≥8 years) was related to a significantly higher volume of the parahippocampal gyrus and thicker left orbital gyri, left precuneus, left superior temporal sulcus, right posterior-dorsal part of the cingulate gyrus, right middle temporal gyrus, and right subparietal sulcus (Table 3). Lengthy mobile phone use (2-4 years, ≥8 years) was related to higher mean FA in the pontine crossing tract on the FA skeleton (Table 4). Often playing computer games was related to a higher volume in the left anterior part of the cingulate gyrus and sulcus and the left triangular part of the inferior frontal gyrus, while sometimes playing computer games was related to a lower volume of the parahippocampal gyrus (Table 3). In addition, often holding a mobile phone on the right side of the head was related to larger areas in the bilateral posterior division of the middle temporal gyrus, bilateral anterior division of the cingulate gyrus, and a larger area of several regions compared to those who usually held their phones on the left side of the head (Table 3). Results of sensitivity analyses of neuroimaging are displayed in Tables S15-S17 in Multimedia Appendix 1.

**Table 3:** Brain areas^a^ where gray matter metrics were related to the use of electronic devices.

Brain areas		β (SE)	*T* value	*P* value
**Mobile phone use>8years (reference: never used a mobile phone at least once per week)**				
	Mean thickness of G-orbital (left)	0.011 (0.003)	3.678	2.35E-04
	Mean thickness of G-precuneus (left)	0.011 (0.003)	3.670	2.43E-04
	Mean thickness of S-temporal-sup (left)	0.009 (0.002)	3.928	8.57E-05
	Volume of G-oc-temp-med-parahip (left)	45.899 (12.370)	3.711	2.07E-04
	Mean thickness of G-cingul-post-dorsal (right)	0.013 (0.003)	3.959	7.56E-05
	Mean thickness of G-temporal-middle (right)	0.011 (0.003)	3.841	1.23E-04
	Mean thickness of S-subparietal (right)	0.012 (0.003)	4.304	1.69E-05
**Mobile phone use=5-8 years**				
	Mean thickness of S-temporal-sup (left)	0.009 (0.002)	3.779	1.57E-04
	Area of G-rectus (right)	–3.918 (0.985)	–3.978	6.96E-05
	Mean thickness of G-oc-temp-lat-fusifor (right)	0.013 (0.003)	3.667	2.45E-04
**Mobile phone use=2-4 years**				
	Mean thickness of S-temporal-sup (left)	0.019 (0.005)	3.616	3.00E-04
	Area of S-orbital-med-olfact (right)	–11.055 (3.020)	–3.661	2.52E-04
**Weekly usage of mobile phones in the past 3 months (reference: <5 minutes)**				
	Area of S-collat-transv-post (right)	–3.934 (1.050)	–3.748	1.79E-04
	Volume of S-collat-transv-post (right)	–10.803 (2.410)	–4.483	7.39E-06
**Mobile phone held usually on the right side of the head (reference: left)**				
	Area of G+S-cingul-mid-ant (left)	6.742 (1.707)	3.950	7.83E-05
	Area of G-cingul-post-dorsal (left)	4.535 (0.883)	5.135	2.84E-07
	Area of G-cingul-post-ventral (left)	1.681 (0.463)	3.628	2.86E-04
	Area of G-front-middle (left)	18.051 (4.991)	3.617	2.98E-04
	Area of S-pericallosal (left)	8.164 (1.941)	4.206	2.61E-05
	Area of S-temporal-sup (left)	20.326 (5.660)	3.591	3.30E-04
	Volume of G-cingul-post-dorsal (left)	13.244 (3.587)	3.692	2.23E-04
	Area of G+S-frontomargin (right)	3.896 (0.995)	3.915	9.05E-05
	Area of G-pariet-inf-angular (right)	14.439 (3.724)	3.877	1.06E-04
	Area of G-temporal-middle (right)	10.537 (2.926)	3.601	3.17E-04
	Area of S-orbital-H-shaped (right)	5.502 (1.458)	3.774	1.61E-04
	Area of S-parieto-occipital (right)	11.220 (2.885)	3.889	1.01E-04
	Area of S-temporal-sup (right)	23.950 (5.787)	4.139	3.50E-05
	Mean thickness of S-front-middle (right)	–0.006 (0.002)	–4.030	5.60E-05
**Plays computer games often (reference: never/rarely)**				
	Volume of G+S-cingul-ant (left)	69.139 (19.050)	3.629	2.85E-04
	Volume of G-front-inf-triangul (left)	59.338 (15.120)	3.925	8.70E-05
**Plays computer games sometimes**				
	Volume of S-postcentral (left)	–45.258 (9.949)	–4.549	5.40E-06
	Volume of G-oc-temp-med-parahip (right)	–33.829 (8.741)	–3.870	1.09E-04
	Area of S-postcentral (left)	–18.319 (4.129)	–4.437	9.16E-06
	Mean thickness of G-Ins-lg+S-cent-ins (left)	–0.014 (0.004)	–3.634	2.79E-04
	Volume of G-Ins-lg+S-cent-ins (left)	–12.194 (3.148)	–3.873	1.08E-04
	Volume of G-oc-temp-med-parahip (left)	–39.076 (9.719)	–4.021	5.81E-05

^a^The full names of the brain areas are listed in the literature [20].

**Table 4:** Brain areas where DTI^a^ metrics were related to the use of electronic devices.

Brain areas		β (SE)	*T* value	*P* value
**Mobile phone use>8years (reference: never used a mobile phone at least once per week)**				
	Mean FA^b^ in the pontine crossing tract	0.003 (0.001)	4.995	5.90E-07
	Mean OD^c^ in posterior thalamic radiation (left)	–0.001 (0.000)	–3.873	1.25E-04
**Mobile phone use=2-4 years**				
	Mean FA in the pontine crossing tract	0.003 (0.001)	3.914	9.11E-05
	Mean OD in the fornix	–0.011 (0.003)	–3.954	7.70E-05
**Weekly usage of mobile phones in the past 3 months (reference: <5 minutes)**				
	Mean OD in the cerebral peduncle (left)	0.001 (0.000)	3.953	7.75E-05
**Mobile phone held usually on the right side of the head (reference: left)**				
	Mean FA in the posterior limb of the internal capsule (right)	0.001 (0.000)	3.741	1.84E-04

^a^DTI: diffusion tensor imaging.

^b^FA: fractional anisotropy.

^c^OD: orientation dispersion.

Overall, the findings of this study showed that electronic device use is associated with a reduced risk of neurodegenerative diseases (Table 5). This table summarizes the results of the main models but not the results of sensitivity analyses.

**Table 5:** Summary of the relationship between electronic device use, risk of disease, and brain structure.

Variables			Risk of ACD^a^		Risk of AD^b^		Risk of VD^c^	Risk of ACP^d^		Risk of PD^e^		Related to brain structure changes
**Usual side of head for mobile phone use**												
	Left (reference)	—^f^		—		—		—	—		—	
	Right	No change		No change		No change		No change	No change		Yes	
	Equally left and right	No change		No change		No change		No change	No change		No	
**Weekly usage of mobile phones in the past 3 months**												
	<5 minutes (reference)	—		—		—		—	—		—	
	≥5 minutes	No change		No change		No change		No change	No change		Yes	
**Difference in mobile phone use compared to 2 years previously**												
	No change (reference)	—		—		—		—	—		—	
	more frequent	No change		No change		No change		No change	No change		No	
	Less frequent	No change		No change		No change		No change	No change		No	
**Plays computer games**												
	Never/rarely (reference)	—		—		—		—	—		—	
	Sometimes	No change		No change		No change		No change	No change		Yes	
	Often	No change		No change		No change		Decreased	Decreased		Yes	
**Hands-free device/speakerphone use with mobile phones in the past 3 months**												
	Never/almost never (reference)	—		—		—		—	—		—	
	Used	No change		No change		No change		No change	No change		No	
**Length of mobile phone use (years)**												
	Never used a mobile phone at least once per week (reference)	—		—		—		—	—		—	
	≤1	No change		No change		No change		No change	No change		No	
	2-4	Decreased		No change		Decreased		No change	No change		Yes	
	5-8	Decreased		Decreased		Decreased		No change	No change		Yes	
	>8	Decreased		No change		Decreased		No change	No change		Yes	

^a^ACD: all-cause dementia.

^b^AD: Alzheimer disease.

^c^VD: vascular dementia.

^d^ACP: all-cause parkinsonism.

^e^PD: Parkinson disease.

^f^Not applicable.

## Discussion

### Principal Findings

In our study, we found evidence suggesting that the use of electronic devices is associated with a reduced risk of neurodegenerative diseases. Specifically, we observed that using mobile phones for 2 or more years is linked to a decreased risk of ACD and VD compared to never/rarely using mobile phones. Upon conducting sensitivity analyses, we confirmed the stability of the association between long mobile phone use and a lower risk of ACD and VD. Moreover, individuals aged 60 years and older appeared to benefit from extended duration of mobile phone use with a reduction in the risk of AD, ACP, and PD. Furthermore, our neuroimaging analysis revealed that various electronic device use habits, including the duration of mobile phone use, the frequency of computer use, and the preferred side of the head for holding mobile phones, were associated with distinct characteristics in the gray matter and WM structures of several brain regions over the follow-up period.

### Comparison With Prior Work

Our findings suggest that using a mobile phone for more than 2 years is associated with a reduced risk of ACD and VD in the middle-aged population, with no significant impact of weekly mobile phone use. Previous studies using UK Biobank data have reported an association between mobile phone use and a lower risk of ACD in individuals over 60 years old [7]. Our study builds upon this knowledge by focusing on specific types of dementia and details of mobile phone use. We found that a long weekly time of mobile phone use (≥5 minutes) does not alter the risk of dementia compared to a shorter weekly time (<5 minutes). However, the duration of mobile phone use affects the association between mobile phone use and reduced risk of dementia. Using a mobile phone enriches the user’s social and mental activities, potentially leading to improved cognition in later life [25,26]. The association between mobile phone use and a decreased risk of dementia is partly mediated by rich social support and flourishing leisure activities [7]. Additionally, prior research has suggested that registering a mobile phone decreases the risk of PD [8]. Our study further revealed that this association is observed only in populations aged 60 years and older, where a long duration of mobile phone use decreases the risk of developing ACP and PD compared to those who rarely use mobile phones.

The benefits of using electronic devices can be reflected in improved brain structure. We found that long durations of mobile phone use are associated with a thicker cortex in multiple areas of the brain. Specifically, individuals who use mobile phones for more than 8 years exhibit a higher volume of the parahippocampal gyrus, a region crucial for memory function [27]. This finding is particularly significant as atrophy in the parahippocampal gyrus is observed in the early stages of AD, serving as an early pathological biomarker [28,29]. Previous studies have shown that daily mobile phone use for less than 1 hour is associated with lower functional connectivity in certain brain regions, such as the superior temporal sulcus, and higher functional connectivity in others, like the cingulate [14]. In this study, we observed that only weekly mobile phone use of 5 minutes or more is associated with a decreased volume and area in the right posterior transverse collateral sulcus compared to less than 5 minutes of weekly use. However, no significant results were found in other areas or the DTI analysis. These findings suggest that daily mobile phone use, without reaching problematic levels, has minimal impact on the user’s brain structure. Interestingly, we found inconsistent effects of occasional or frequent computer usage on the brain structure. Often playing computer games is associated with a higher brain volume, while sometimes playing computer games is related to a lower brain volume. For those who sometimes play computers, the related shortcomings, such as sedentariness, are more prominent and result in cortical atrophy [30].

### Strengths and Limitations

This study has several strengths. First, we adjusted for covariates that may have influenced the results using comprehensive data from the UK Biobank, which is the largest population-based cohort in the world. This allowed us to provide important information on the use of electronic devices. Second, the cohort’s follow-up duration was notably extensive, allowing sufficient time for patients to reach the point at which diseases developed. Additionally, the duration between baseline and neuroimaging scans offered crucial insights into the long-term effects of electronic device use on brain health.

There was 1 limitation that should be mentioned. The mobile phone use in this study was limited to receiving calls. The rapid advancement and evolution of electronic devices in recent years have transformed the usage patterns of mobile phones, heightening the potential for dependency [31,32]. However, the evolution happened years after the launch of the UK Biobank cohort. Our study still provides important insights into the long-term influence of electronic device use. The time that people spend on mobile phones now is much longer than it was decades before [31,33]. However, this study found that there are no additional benefits of longer weekly usage. In addition, playing games on the mobile phone is popular nowadays [33]. We found an association between playing computer games and decreased neurodegenerative disease risk, but the association is unclear if game devices change from computers to mobile phones. Future studies should record the details of the daily time and the type of function of mobile phone use to analyze the impact of modern electronic devices on brain health.

### Conclusion

In conclusion, our study revealed that individuals with long duration of mobile phone use are associated with a decreased risk of ACD and VD compared to those who rarely use mobile phones. The protective effect of mobile phone use is also observed in individuals older than 60 years, who experience a reduced risk of AD, ACP, and PD. However, spending more time on mobile phones per week may not further reduce the risk of neurodegenerative disease. Our findings also indicate that electronic device use can impact the brain structure. Specifically, using a mobile phone and often playing computer games are associated with a better brain structure, while sometimes playing computer games is linked to a poorer brain structure. These results highlight the complex relationship between electronic device use and neurodegenerative disease risk, as well as the influence on the brain structure. Future studies should concentrate on the mechanism underlying the influence of using mobile phones and computers and new electronic devices on health.

## Appendix

Multimedia Appendix 1 Demographic characteristics of participants and association between the risk of neurodegenerative diseases and electronic device use.

## Abbreviations

ACD: all-cause dementia
ACP: all-cause parkinsonism
AD: Alzheimer disease
DTI: diffusion tensor imaging
FA: fractional anisotropy
HR: hazard ratio
ICVF: intracellular volume fraction
ISOVF: isotropic (free) water volume fraction
MD: mean diffusion
MRI: magnetic resonance imaging
OD: orientation dispersion
PD: Parkinson disease
VD: vascular dementia
WM: white matter

## Data Availability

All code is available upon reasonable request.
