# Supplementary material for: Long-Term Impact of Using Mobile Phones and Playing Computer Games on the Brain Structure and the Risk of Neurodegenerative Diseases: Large Population-Based Study
Source: J Med Internet Res. 2025 Jan 28;27:e59663. doi: 10.2196/59663 (PMC11815302; doi:10.2196/59663)
Supplement: Multimedia Appendix 1 [file jmir_v27i1e59663_app1.docx]

# Multimedia Appendix 1

## Covariates

Description of the electronic device use questionnaire:

| Field ID | Name | Question | Description of choice of answer | Covariate in the modules |
| --- | --- | --- | --- | --- |
| 1110 | Length of mobile phone use | For approximately how many years have you been using a mobile phone at least once per week to make or receive calls | Never used a mobile phone at least once per week  One year or less  Two to four years  Five to eight years  More than eight years  Do not know  Prefer not to answer | Category (5 levels) |
| 1120 | Weekly usage of mobile phone in last 3 months | Over the last 3 months, on average how much time per week did you spend making or receiving calls on a mobile phone? | Less than 5mins  5-29 mins  30-59 mins  1-3 hours  4-6 hours  More than 6 hours  Do not know  Prefer not to answer | Category (2 levels: less than 5 minutes; ≥ 5 minutes) |
| 1130 | Hands-free device/speakerphone use with mobile phone in last 3 months | Over the last 3 months, how often have you used a hands-free device/speakerphone when making or receiving calls on your mobile? | Never or almost never  Less than half the time  About half the time  More than half the time  Always or almost always  Do not know  Prefer not to answer | Category (2 levels: never /almost never; used) |
| 1140 | Difference in mobile phone use compared to two years previously | Is there any difference between your mobile phone use now compared to two years ago? | No  Yes, use is now less frequent  Yes, use is now more frequent  I didn’t use a mobile phone two years ago  Do not know  Prefer not to answer | Category (3 levels: no change (including person that didn’t use a mobile phone two years ago); more frequent; less frequent) |
| 1150 | Usual side of the head for mobile phone use | On what side of the head do you usually use a mobile phone? | Left  Right  Equally left and right  Do not know  Prefer not to answer | Category (3 levels) |
| 2237 | Plays computer games | Do you play computer games? | Never/rarely  Sometimes  Often  Prefer not to answer | Category (3 levels) |

PS: Do not know and prefer not to answer were classified as the same level or excluded from the classification of variates.

Field ID of the covariates

| Variates | Field ID |
| --- | --- |
| Age | 21022 |
| sex | 31 |
| Body mass index | 21001 |
| smoking | 20116 |
| drinking | 20117 |
| hypertension | Combine 131286,131287 |
| diabetes | From 130706 to 130715 |
| Education | 21001 |
| polygenic risk scores | 20206 for dementia; 26260 for Parkinsonism |
| socioeconomic status | 189 |
| average total household income before tax | 738 |
| Total volume of white matter hyperintensities | 25781 |
| The volume of white matter | 25007 |
| The volume of grey matter | 25005 |
| Mental health score | The higher the number, the more symptoms are associated with poor mental health. The sum score of the data fields of interest included (1) UKB  mood swings (Field ID:1920; Does your mood often go up and down?), (2) UKB  miserableness (Field ID:1930; Do you ever feel 'just miserable' for no reason?), (3) UKB  irritability (Field ID:1940; Are you an irritable person?), (4) UKB sensitivity / hurt feelings  (Field ID:1950; Are your feelings easily hurt ?), (5) UKB fed-up feelings (Field ID:1960; Do  you often feel 'fed-up'?), (6) UKB nervous feelings (Field ID:1970; Would you call yourself a  nervous person?), (7) UKB worrier / anxious feelings (Field ID:1980; Are you a worrier?),  (8) UKB tense / 'highly strung' (Field ID:1990; Would you call yourself tense or 'highly  strung'?), (9) UKB worry too long after embarrassment (Field ID:2000; Do you worry too  long after an embarrassing experience?), (10) UKB suffer from 'nerves' (Field ID:2010; Do  you suffer from 'nerves'?), (11) UKB loneliness, isolation (Field ID:2020; Do you often feel  lonely?), (12) UKB guilty feelings (Field ID:2030; Are you often troubled by feelings of  guilt?), (13) UKB risk taking (Field ID:2040; Would you describe yourself as someone who  takes risks?). Questions to all answers coded as ‘yes’, ‘no’, ‘do not know’, ‘prefer not to  answer’ |
| Regular physical activity | Regular physical activity criteria was defined as meeting the recommendations of at least 75 minutes of moderate activity or 25 minutes of vigorous activity per week. (field ID: 22039/22038) |

## Neuroimage

The mechanism of DTI is based on the anisotropy that water molecules spread faster in the major axis of the fiber than in other directions. DTI metrics fractional anisotropy (FA) and mean diffusion (MD) indicate the overall integrity of WM fiber [1]. Recently, a novel diffusion tensor model, neurite orientation dispersion and density imaging (NODDI), and its derived metrics were proposed, expanding the ability of diffusion MRI [2]. NODDI disentangles the key contributors of the FA, providing independent estimates of neurite density and orientation dispersion. DTI was able to detect the cognitive impairment-related microinjury [3].

**References**

1. Basser, P. J. Inferring microstructural features and the physiological state of tissues from diffusion-weighted images. *NMR Biomed* **8**, 333-344, doi:10.1002/nbm.1940080707 (1995).
2. Zhang, H., Schneider, T., Wheeler-Kingshott, C. A. & Alexander, D. C. NODDI: practical in vivo neurite orientation dispersion and density imaging of the human brain. *Neuroimage* **61**, 1000-1016, doi:10.1016/j.neuroimage.2012.03.072 (2012).
3. Chandra, A., Dervenoulas, G., Politis, M. & Alzheimer's Disease Neuroimaging, I. Magnetic resonance imaging in Alzheimer's disease and mild cognitive impairment. *Journal of neurology* **266**, 1293-1302, doi:10.1007/s00415-018-9016-3 (2019).

## Inclusion flowchart of Cox analysis (detailed version)


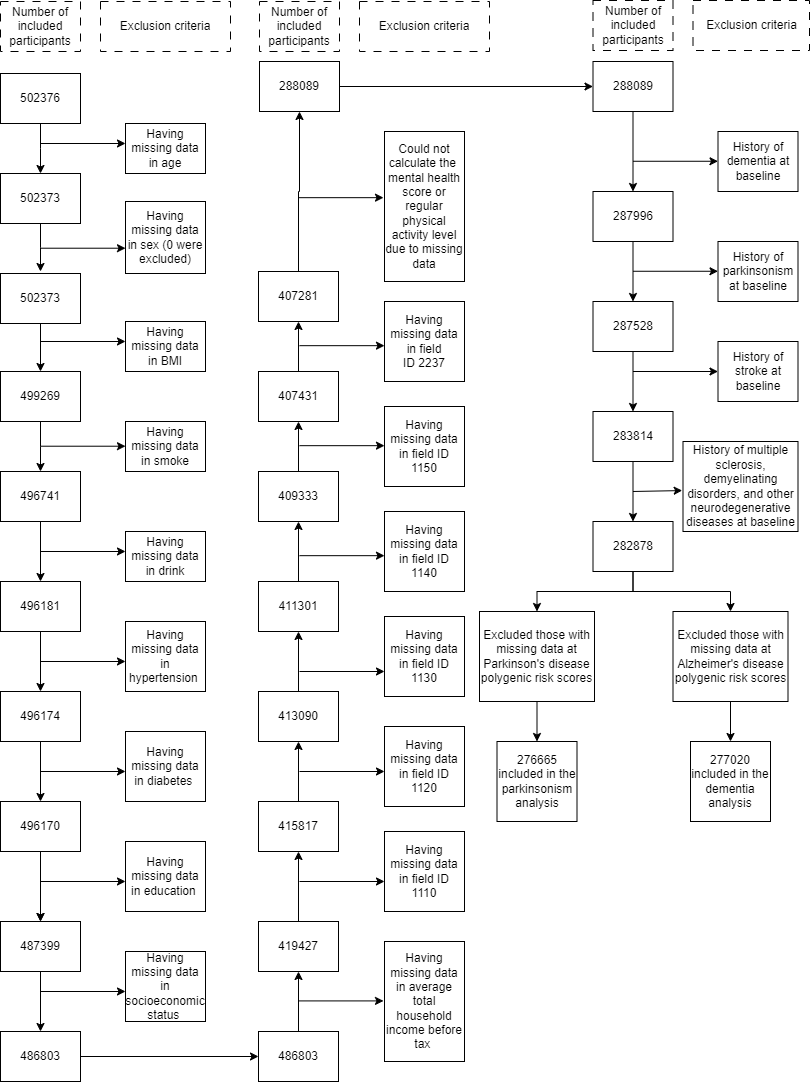


|  | Table S1. Demographic characteristics of participants included and excluded from the Parkinsonism risk analysis. | | | | | |
| --- | --- | --- | --- | --- | --- | --- |
| **Variable** | | **Overall, N = 502,376^a^** | **Excluded, N = 225,711^a^** | **Included, N = 276,665^a^** | **Test value^b^** | ***P*-value** |
| Usual side of head for mobile phone use | |  |  |  | 298.05 | <.001 |
| Left | | 149,338 (36%) | 65,259 (36%) | 84,079 (35%) |  |  |
| Right | | 241,652 (58%) | 102,377 (56%) | 139,275 (58%) |  |  |
| Equally left and right | | 28,784 (7%) | 13,665 (8%) | 15,119 (6%) |  |  |
| Do not need to answer | | 82,602 | 44,410 | 38,192 |  |  |
| Weekly usage of mobile phone in last 3 months | | |  |  | 843.77 | <.001 |
| less than 5 mins | | 88,486 (21%) | 41,625 (23%) | 46,861 (20%) |  |  |
| ≥ 5 mins | | 328,086 (79%) | 136,474 (77%) | 191,612 (80%) |  |  |
| Do not need to answer | | 85,804 | 47,612 | 38,192 |  |  |
| Difference in mobile phone use compared to two years previously | | |  |  | 624.74 | <.001 |
| no change | | 225,028 (54%) | 100,684 (56%) | 124,344 (52%) |  |  |
| more frequent | | 54,211 (13%) | 22,660 (13%) | 31,551 (13%) |  |  |
| less frequent | | 139,133 (33%) | 56,555 (31%) | 82,578 (35%) |  |  |
| Do not need to answer | | 84,004 | 45,812 | 38,192 |  |  |
| Plays computer games | |  |  |  | 1.48 | .48 |
| Never/rarely | | 395,277 (79%) | 176,691 (79%) | 218,586 (79%) |  |  |
| Sometimes | | 88,607 (18%) | 39,746 (18%) | 48,861 (18%) |  |  |
| Often | | 16,782 (3%) | 7,564 (3%) | 9,218 (3%) |  |  |
| Unknown | | 1,710 | 1,710 | 0 |  |  |
| Hands-free device/speakerphone use with mobile phone in last 3 month | | | |  | 1420.65 | <.001 |
| never/almost never | | 344,600 (82%) | 152,775 (85%) | 191,825 (80%) |  |  |
| used | | 73,767 (18%) | 27,119 (15%) | 46,648 (20%) |  |  |
| Do not need to answer | | 84,009 | 45,817 | 38,192 |  |  |
| Length of mobile phone use | |  |  |  | 3786.84 | <.001 |
| Never used mobile phone at least once per week | | 73,809 (15%) | 35,617 (16%) | 38,192 (14%) |  |  |
| One year or less | | 13,666 (3%) | 7,350 (3%) | 6,316 (2%) |  |  |
| Two to four years | | 87,154 (18%) | 42,708 (20%) | 44,446 (16%) |  |  |
| Five to eight years | | 152,091 (31%) | 66,931 (31%) | 85,160 (31%) |  |  |
| More than eight years | | 167,607 (34%) | 65,056 (30%) | 102,551 (37%) |  |  |
| Unknown | | 8,049 | 8,049 | 0 |  |  |
| **age** | | 56.53±8.09 | 57.36±8.05 | 55.85±8.07 | 65.81 | <.001 |
| Unknown | | 3 | 3 | 0 |  |  |
| **sex** | |  |  |  | 4175.00 | <.001 |
| female | | 273,305 (54%) | 134,139 (59%) | 139,166 (50%) |  |  |
| male | | 229,071 (46%) | 91,572 (41%) | 137,499 (50%) |  |  |
| **BMI** | | 27.43±4.80 | 27.62±4.98 | 27.29±4.65 | 24.07 | <.001 |
| Unknown | | 3,107 | 3,107 | 0 |  |  |
| **smoke** | |  |  |  | 3.24 | .071 |
| Yes | | 273,453 (55%) | 121,654 (55%) | 151,799 (55%) |  |  |
| No | | 225,973 (45%) | 101,107 (45%) | 124,866 (45%) |  |  |
| Unknown | | 2,950 | 2,950 | 0 |  |  |
| **drink** | |  |  |  | 1910.29 | <.001 |
| Yes | | 22,379 (4%) | 13,192 (6%) | 9,187 (3%) |  |  |
| No | | 478,343 (96%) | 210,865 (94%) | 267,478 (97%) |  |  |
| Unknown | | 1,654 | 1,654 | 0 |  |  |
| **hypertension** | |  |  |  | 752.80 | <.001 |
| Yes | | 134,455 (27%) | 64,691 (29%) | 69,764 (25%) |  |  |
| No | | 367,914 (73%) | 161,013 (71%) | 206,901 (75%) |  |  |
| Unknown | | 7 | 7 | 0 |  |  |
| **all diabetes status** | |  |  |  | 536.50 | <.001 |
| Yes | | 476,101 (95%) | 212,083 (94%) | 264,018 (95%) |  |  |
| No | | 26,266 (5%) | 13,619 (6%) | 12,647 (5%) |  |  |
| Unknown | | 9 | 9 | 0 |  |  |
| **education** | |  |  |  | 7324.41 | <.001 |
| college/university degree | | 331,135 (67%) | 159,000 (74%) | 172,135 (62%) |  |  |
| Lower degree | | 161,109 (33%) | 56,579 (26%) | 104,530 (38%) |  |  |
| Unknown | | 10,132 | 10,132 | 0 |  |  |
| **Parkinson's disease polygenic risk scores** | | -0.14±1.02 | -0.14±1.02 | -0.14±1.02 | 2.58 | .018 |
| Unknown | | 16,243 | 16,243 | 0 |  |  |
| Townsend deprivation index | | -1.29±3.09 | -1.04±3.23 | -1.50±2.97 | 52.17 | <.001 |
| Unknown | | 626 | 626 | 0 |  |  |
| Average Total Household Income Before Tax | |  |  |  | 9199.20 | <.001 |
| 18,000 To 30,999 | | 97,177 (23%) | 43,801 (29%) | 53,376 (19%) |  |  |
| 31,000 To 51,999 | | 108,144 (25%) | 40,680 (27%) | 67,464 (24%) |  |  |
| 52,000 To 100,000 | | 110,747 (26%) | 35,817 (24%) | 74,930 (27%) |  |  |
| Do Not Know | | 86,244 (20%) | 23,272 (16%) | 62,972 (23%) |  |  |
| Greater Than 100,000 | | 22,923 (5%) | 5,000 (3%) | 17,923 (6%) |  |  |
| Unknown | | 77,141 | 77,141 | 0 |  |  |
| emotional score | | 4.40±3.28 | 4.61±3.33 | 4.31±3.25 | 26.22 | <.001 |
| Unknown | | 108,401 | 108,401 | 0 |  |  |
| regulart PA | |  |  |  | 541.36 | <.001 |
| not meeting the criteria | | 63,507 (16%) | 22,324 (18%) | 41,183 (15%) |  |  |
| meeting the criteria | | 338,768 (84%) | 103,286 (82%) | 235,482 (85%) |  |  |
| Unknown | | 100,101 | 100,101 | 0 |  |  |
| ^a^Mean±SD or Frequency (%) | |  |  |  |  |  |
| ^b^Test value for continuous variables was the T-test value and the test value for category variables was the test value of the chi-square test or Fisher exact test. | |  |  |  |  |  |

| Table S2. Demographic characteristics of participants included and excluded from the dementia risk analysis. | | | | |  |  |
| --- | --- | --- | --- | --- | --- | --- |
| **Variable** | | **Overall**, N = 502,376^a^ | Excluded, N = 225,356^a^ | Included, N = 277,020^a^ | **Test value^b^** | ***P*-value** |
| Usual side of head for mobile phone use | |  |  |  | 300.09 | <.001 |
| Left | | 149,338 (36%) | 65,157 (36%) | 84,181 (35%) |  |  |
| Right | | 241,652 (58%) | 102,223 (56%) | 139,429 (58%) |  |  |
| Equally left and right | | 28,784 (7%) | 13,653 (8%) | 15,131 (6%) |  |  |
| Do not need to answer | | 82,602 | 44,323 | 38,279 |  |  |
| Weekly usage of mobile phone in last 3 months | |  |  |  | 834.74 | <.001 |
| less than 5 mins | | 88,486 (21%) | 41,547 (23%) | 46,939 (20%) |  |  |
| ≥ 5 mins | | 328,086 (79%) | 136,284 (77%) | 191,802 (80%) |  |  |
| Do not need to answer | | 85,804 | 47,525 | 38,279 |  |  |
| Difference in mobile phone use compared to two years previously | | |  |  | 621.03 | <.001 |
| no change | | 225,028 (54%) | 100,532 (56%) | 124,496 (52%) |  |  |
| more frequenct | | 54,211 (13%) | 22,611 (13%) | 31,600 (13%) |  |  |
| less frequent | | 139,133 (33%) | 56,488 (31%) | 82,645 (35%) |  |  |
| Do not need to answer | | 84,004 | 45,725 | 38,279 |  |  |
| Plays computer games | |  |  |  | 1.54 | .46 |
| Never/rarely | | 395,277 (79%) | 176,404 (79%) | 218,873 (79%) |  |  |
| Sometimes | | 88,607 (18%) | 39,692 (18%) | 48,915 (18%) |  |  |
| Often | | 16,782 (3%) | 7,550 (3%) | 9,232 (3%) |  |  |
| Unknown | | 1,710 | 1,710 | 0 |  |  |
| Hands-free device/speakerphone use with mobile phone in last 3 months | | |  |  | 1414.22 | <.001 |
| never/almost never | | 344,600 (82%) | 152,543 (85%) | 192,057 (80%) |  |  |
| used | | 73,767 (18%) | 27,083 (15%) | 46,684 (20%) |  |  |
| Do not need to answer | | 84,009 | 45,730 | 38,279 |  |  |
| Length of mobile phone use | |  |  |  | 3767.12 | <.001 |
| Never used mobile phone at least once per week | | 73,809 (15%) | 35,530 (16%) | 38,279 (14%) |  |  |
| One year or less | | 13,666 (3%) | 7,341 (3%) | 6,325 (2%) |  |  |
| Two to four years | | 87,154 (18%) | 42,647 (20%) | 44,507 (16%) |  |  |
| Five to eight years | | 152,091 (31%) | 66,828 (31%) | 85,263 (31%) |  |  |
| More than eight years | | 167,607 (34%) | 64,961 (30%) | 102,646 (37%) |  |  |
| Unknown | | 8,049 | 8,049 | 0 |  |  |
| **age** | | 56.53±8.09 | 57.35±8.05 | 55.86±8.07 | 65.19 | <.001 |
| Unknown | | 3 | 3 | 0 |  |  |
| **sex** | |  |  |  | 4237.64 | <.001 |
| female | | 273,305 (54%) | 134,029 (59%) | 139,276 (50%) |  |  |
| male | | 229,071 (46%) | 91,327 (41%) | 137,744 (50%) |  |  |
| **BMI** | | 27.43±4.80 | 27.62±4.98 | 27.29±4.65 | 24.10 | <.001 |
| Unknown | | 3,107 | 3,107 | 0 |  |  |
| **smoke** | |  |  |  | 3.86 | .049 |
| Yes | | 273,453 (55%) | 121,431 (55%) | 152,022 (55%) |  |  |
| No | | 225,973 (45%) | 100,975 (45%) | 124,998 (45%) |  |  |
| Unknown | | 2,950 | 2,950 | 0 |  |  |
| **drink** | |  |  |  | 1916.73 | <.001 |
| Yes | | 22,379 (4%) | 13,181 (6%) | 9,198 (3%) |  |  |
| No | | 478,343 (96%) | 210,521 (94%) | 267,822 (97%) |  |  |
| Unknown | | 1,654 | 1,654 | 0 |  |  |
| **hypertension** | |  |  |  | 751.61 | <.001 |
| Yes | | 134,455 (27%) | 64,592 (29%) | 69,863 (25%) |  |  |
| No | | 367,914 (73%) | 160,757 (71%) | 207,157 (75%) |  |  |
| Unknown | | 7 | 7 | 0 |  |  |
| **all diabetes status** | |  |  |  | 538.76 | <.001 |
| Yes | | 476,101 (95%) | 211,743 (94%) | 264,358 (95%) |  |  |
| No | | 26,266 (5%) | 13,604 (6%) | 12,662 (5%) |  |  |
| Unknown | | 9 | 9 | 0 |  |  |
| **education** | |  |  |  | 7337.37 | <.001 |
| college/university degree | | 331,135 (67%) | 158,771 (74%) | 172,364 (62%) |  |  |
| Lower degree | | 161,109 (33%) | 56,453 (26%) | 104,656 (38%) |  |  |
| Unknown | | 10,132 | 10,132 | 0 |  |  |
| **Alzheimer's disease polygenic risk scores** | | 0.05±1.00 | 0.05±1.00 | 0.05±0.99 | 1.96 | .085 |
| Unknown | | 16,243 | 16,243 | 0 |  |  |
| Townsend deprivation index | | -1.29±3.09 | -1.04±3.23 | -1.50±2.97 | 52.36 | <.001 |
| Unknown | | 626 | 626 | 0 |  |  |
| Average Total Household Income Before Tax | |  |  |  | 9144.6 | <.001 |
| 18,000 To 30,999 | | 97,177 (23%) | 43,692 (29%) | 53,485 (19%) |  |  |
| 31,000 To 51,999 | | 108,144 (25%) | 40,565 (27%) | 67,579 (24%) |  |  |
| 52,000 To 100,000 | | 110,747 (26%) | 35,736 (24%) | 75,011 (27%) |  |  |
| Do Not Know | | 86,244 (20%) | 23,235 (16%) | 63,009 (23%) |  |  |
| Greater Than 100,000 | | 22,923 (5%) | 4,987 (3%) | 17,936 (6%) |  |  |
| Unknown | | 77,141 | 77,141 | 0 |  |  |
| emotional score | | 4.40±3.28 | 4.61±3.33 | 4.31±3.25 | 26.31 | <.001 |
| Unknown | | 108,401 | 108,401 | 0 |  |  |
| regulart PA | |  |  |  | 534.85 | <.001 |
| not meeting the criteria | | 63,507 (16%) | 22,251 (18%) | 41,256 (15%) |  |  |
| meeting the criteria | | 338,768 (84%) | 103,004 (82%) | 235,764 (85%) |  |  |
| Unknown | | 100,101 | 100,101 | 0 |  |  |
|  | ^a^Mean±SD or Frequency (%) | | | | | |
|  | ^b^Test value for continuous variables was T-test value and the test value for category variables wa test value of chi-square test or Fisher exact test | | | | | |

|  | Table S3. The association between the risk of dementia and electronic device use. | | | | | | | | |  | |  |
| --- | --- | --- | --- | --- | --- | --- | --- | --- | --- | --- | --- | --- |
| Variables | | HR | 95%CI lower | 95%CI upper | Number of outcomes | Total | Z value | *P value^a^* | outcome | | exposure | |
| Left | | 1.000 | 1.000 | 1.000 | 833 | 84181 |  |  | all-cause dementia | | Usual side of head for mobile phone use | |
| Right | | 1.027 | 0.941 | 1.121 | 1255 | 139429 | 0.589 | 5.56E-01 | all-cause dementia | | Usual side of head for mobile phone use | |
| Equally left and right | | 1.041 | 0.863 | 1.256 | 126 | 15131 | 0.418 | 6.76E-01 | all-cause dementia | | Usual side of head for mobile phone use | |
| less than 5 mins | | 1.000 | 1.000 | 1.000 | 596 | 46939 |  |  | all-cause dementia | | Weekly usage of mobile phone in last 3 months | |
| ≥ 5 mins | | 1.086 | 0.987 | 1.194 | 1618 | 191802 | 1.689 | 9.12E-02 | all-cause dementia | | Weekly usage of mobile phone in last 3 months | |
| no change | | 1.000 | 1.000 | 1.000 | 1301 | 124496 |  |  | all-cause dementia | | Difference in mobile phone use compared to two years previously | |
| more frequenct | | 1.114 | 0.993 | 1.250 | 383 | 31600 | 1.833 | 6.69E-02 | all-cause dementia | | Difference in mobile phone use compared to two years previously | |
| less frequent | | 0.951 | 0.858 | 1.053 | 530 | 82645 | -0.969 | 3.33E-01 | all-cause dementia | | Difference in mobile phone use compared to two years previously | |
| Never/rarely | | 1.000 | 1.000 | 1.000 | 2462 | 218873 |  |  | all-cause dementia | | Plays computer games | |
| Sometimes | | 0.972 | 0.878 | 1.076 | 444 | 48915 | -0.541 | 5.89E-01 | all-cause dementia | | Plays computer games | |
| Often | | 0.995 | 0.815 | 1.215 | 101 | 9232 | -0.045 | 9.64E-01 | all-cause dementia | | Plays computer games | |
| never/almost never | | 1.000 | 1.000 | 1.000 | 1951 | 192057 |  |  | all-cause dementia | | Hands-free device/speakerphone use with mobile phone in last 3 month | |
| used | | 1.005 | 0.879 | 1.148 | 263 | 46684 | 0.070 | 9.44E-01 | all-cause dementia | | Hands-free device/speakerphone use with mobile phone in last 3 months | |
| Never used mobile phone at least once per week | | 1.000 | 1.000 | 1.000 | 793 | 38279 |  |  | all-cause dementia | | Length of mobile phone use | |
| One year or less | | 1.002 | 0.824 | 1.219 | 115 | 6325 | 0.022 | 9.82E-01 | all-cause dementia | | Length of mobile phone use | |
| Two to four years | | 0.815 | 0.729 | 0.912 | 510 | 44507 | -3.566 | **3.62E-04** | all-cause dementia | | Length of mobile phone use | |
| Five to eight years | | 0.749 | 0.677 | 0.829 | 750 | 85263 | -5.600 | **2.15E-08** | all-cause dementia | | Length of mobile phone use | |
| More than eight years | | 0.830 | 0.751 | 0.918 | 839 | 102646 | -3.630 | **2.84E-04** | all-cause dementia | | Length of mobile phone use | |
| Left | | 1.000 | 1.000 | 1.000 | 341 | 84181 |  |  | Alzheimer’s disease | | Usual side of head for mobile phone use | |
| Right | | 1.023 | 0.892 | 1.173 | 513 | 139429 | 0.328 | 7.43E-01 | Alzheimer’s disease | | Usual side of head for mobile phone use | |
| Equally left and right | | 1.159 | 0.873 | 1.538 | 56 | 15131 | 1.020 | 3.08E-01 | Alzheimer’s disease | | Usual side of head for mobile phone use | |
| less than 5 mins | | 1.000 | 1.000 | 1.000 | 243 | 46939 |  |  | Alzheimer’s disease | | Weekly usage of mobile phone in last 3 months | |
| ≥ 5 mins | | 1.144 | 0.986 | 1.328 | 667 | 191802 | 1.770 | 7.67E-02 | Alzheimer’s disease | | Weekly usage of mobile phone in last 3 months | |
| no change | | 1.000 | 1.000 | 1.000 | 530 | 124496 |  |  | Alzheimer’s disease | | Difference in mobile phone use compared to two years previously | |
| more frequenct | | 1.240 | 1.039 | 1.481 | 165 | 31600 | 2.384 | 1.71E-02 | Alzheimer’s disease | | Difference in mobile phone use compared to two years previously | |
| less frequent | | 0.972 | 0.828 | 1.141 | 215 | 82645 | -0.344 | 7.31E-01 | Alzheimer’s disease | | Difference in mobile phone use compared to two years previously | |
| Never/rarely | | 1.000 | 1.000 | 1.000 | 1027 | 218873 |  |  | Alzheimer’s disease | | Plays computer games | |
| Sometimes | | 0.918 | 0.779 | 1.081 | 169 | 48915 | -1.026 | 3.05E-01 | Alzheimer’s disease | | Plays computer games | |
| Often | | 0.855 | 0.610 | 1.199 | 35 | 9232 | -0.906 | 3.65E-01 | Alzheimer’s disease | | Plays computer games | |
| never/almost never | | 1.000 | 1.000 | 1.000 | 808 | 192057 |  |  | Alzheimer’s disease | | Hands-free device/speakerphone use with mobile phone in last 3 month | |
| used | | 1.044 | 0.844 | 1.292 | 102 | 46684 | 0.400 | 6.89E-01 | Alzheimer’s disease | | Hands-free device/speakerphone use with mobile phone in last 3 month | |
| Never used mobile phone at least once per week | | 1.000 | 1.000 | 1.000 | 321 | 38279 |  |  | Alzheimer’s disease | | Length of mobile phone use | |
| One year or less | | 1.151 | 0.860 | 1.540 | 53 | 6325 | 0.945 | 3.45E-01 | Alzheimer’s disease | | Length of mobile phone use | |
| Two to four years | | 0.919 | 0.774 | 1.091 | 229 | 44507 | -0.968 | 3.33E-01 | Alzheimer’s disease | | Length of mobile phone use | |
| Five to eight years | | 0.787 | 0.672 | 0.922 | 311 | 85263 | -2.973 | **2.95E-03** | Alzheimer’s disease | | Length of mobile phone use | |
| More than eight years | | 0.835 | 0.711 | 0.979 | 317 | 102646 | -2.215 | 2.68E-02 | Alzheimer’s disease | | Length of mobile phone use | |
| Left | | 1.000 | 1.000 | 1.000 | 167 | 84181 |  |  | vascular dementia | | Usual side of head for mobile phone use | |
| Right | | 1.035 | 0.851 | 1.259 | 249 | 139429 | 0.343 | 7.32E-01 | vascular dementia | | Usual side of head for mobile phone use | |
| Equally left and right | | 1.218 | 0.820 | 1.809 | 29 | 15131 | 0.979 | 3.28E-01 | vascular dementia | | Usual side of head for mobile phone use | |
| less than 5 mins | | 1.000 | 1.000 | 1.000 | 125 | 46939 |  |  | vascular dementia | | Weekly usage of mobile phone in last 3 months | |
| ≥ 5 mins | | 1.062 | 0.861 | 1.310 | 320 | 191802 | 0.559 | 5.76E-01 | vascular dementia | | Weekly usage of mobile phone in last 3 months | |
| no change | | 1.000 | 1.000 | 1.000 | 276 | 124496 |  |  | vascular dementia | | Difference in mobile phone use compared to two years previously | |
| more frequenct | | 1.001 | 0.776 | 1.290 | 78 | 31600 | 0.005 | 9.96E-01 | vascular dementia | | Difference in mobile phone use compared to two years previously | |
| less frequent | | 0.832 | 0.655 | 1.057 | 91 | 82645 | -1.504 | 1.33E-01 | vascular dementia | | Difference in mobile phone use compared to two years previously | |
| Never/rarely | | 1.000 | 1.000 | 1.000 | 510 | 218873 |  |  | vascular dementia | | Plays computer games | |
| Sometimes | | 1.022 | 0.822 | 1.271 | 98 | 48915 | 0.198 | 8.43E-01 | vascular dementia | | Plays computer games | |
| Often | | 1.059 | 0.696 | 1.610 | 23 | 9232 | 0.267 | 7.89E-01 | vascular dementia | | Plays computer games | |
| never/almost never | | 1.000 | 1.000 | 1.000 | 397 | 192057 |  |  | vascular dementia | | Hands-free device/speakerphone use with mobile phone in last 3 month | |
| used | | 0.912 | 0.670 | 1.242 | 48 | 46684 | -0.585 | 5.59E-01 | vascular dementia | | Hands-free device/speakerphone use with mobile phone in last 3 month | |
| Never used mobile phone at least once per week | | 1.000 | 1.000 | 1.000 | 186 | 38279 |  |  | vascular dementia | | Length of mobile phone use | |
| One year or less | | 0.832 | 0.539 | 1.284 | 23 | 6325 | -0.830 | 4.07E-01 | vascular dementia | | Length of mobile phone use | |
| Two to four years | | 0.616 | 0.477 | 0.794 | 89 | 44507 | -3.738 | **1.86E-04** | vascular dementia | | Length of mobile phone use | |
| Five to eight years | | 0.729 | 0.589 | 0.902 | 163 | 85263 | -2.910 | **3.62E-03** | vascular dementia | | Length of mobile phone use | |
| More than eight years | | 0.750 | 0.605 | 0.930 | 170 | 102646 | -2.619 | **8.83E-03** | vascular dementia | | Length of mobile phone use | |

95%CI: 95% confidence interval

^a^Significant at level P value ＜ .05/5

|  | Table S4. The association between the risk of dementia and length of mobile phone use (excluded those who had outcomes within five years after baseline). | | | | | | | |  |
| --- | --- | --- | --- | --- | --- | --- | --- | --- | --- |
|  | | HR | 95%CI lower | 95%CI upper | Number of outcomes | Total | Z value | *P^b^* | outcome |
| Reference^a^ | | 1.000 | 1.000 | 1.000 | 718 | 38204 |  |  | all-cause dementia |
| One year or less | | 0.982 | 0.797 | 1.208 | 102 | 6312 | -0.175 | 8.61E-01 | all-cause dementia |
| Two to four years | | 0.830 | 0.738 | 0.934 | 471 | 44468 | -3.108 | **1.88E-03** | all-cause dementia |
| Five to eight years | | 0.751 | 0.675 | 0.835 | 682 | 85195 | -5.290 | **1.22E-07** | all-cause dementia |
| More than eight years | | 0.839 | 0.755 | 0.932 | 769 | 102576 | -3.272 | **1.07E-03** | all-cause dementia |
| Reference^a^ | | 1.000 | 1.000 | 1.000 | 298 | 38204 |  |  | Alzheimer’s disease |
| One year or less | | 1.138 | 0.841 | 1.541 | 49 | 6312 | 0.839 | 4.02E-01 | Alzheimer’s disease |
| Two to four years | | 0.903 | 0.755 | 1.079 | 211 | 44468 | -1.128 | 2.59E-01 | Alzheimer’s disease |
| Five to eight years | | 0.764 | 0.648 | 0.901 | 283 | 85195 | -3.202 | **1.36E-03** | Alzheimer’s disease |
| More than eight years | | 0.821 | 0.695 | 0.969 | 292 | 102576 | -2.327 | 2.00E-02 | Alzheimer’s disease |
| Reference^a^ | | 1.000 | 1.000 | 1.000 | 159 | 38204 |  |  | vascular dementia |
| One year or less | | 0.762 | 0.467 | 1.241 | 18 | 6312 | -1.094 | 2.74E-01 | vascular dementia |
| Two to four years | | 0.651 | 0.497 | 0.852 | 81 | 44468 | -3.124 | **1.78E-03** | vascular dementia |
| Five to eight years | | 0.762 | 0.607 | 0.956 | 147 | 85195 | -2.346 | 1.90E-02 | vascular dementia |
| More than eight years | | 0.789 | 0.628 | 0.992 | 154 | 102576 | -2.026 | 4.27E-02 | vascular dementia |

^a^ The reference level was never used mobile phone at least once per week.

95%CI: 95% confidence interval

^b^Significant at level P value ＜ .05/5

|  | Table S5. The association between the risk of dementia and length of mobile phone use (excluded participants whose source of outcomes were not from the hospital or the death registration). | | | | | | | | |
| --- | --- | --- | --- | --- | --- | --- | --- | --- | --- |
|  | | HR | 95%CI lower | 95%CI upper | Number of outcomes | Total | Z value | *P*^b^ | outcome |
| Reference^a^ | | 1.000 | 1.000 | 1.000 | 793 | 38279 |  |  | all-cause dementia |
| One year or less | | 1.002 | 0.824 | 1.219 | 115 | 6325 | 0.023 | 9.81E-01 | all-cause dementia |
| Two to four years | | 0.814 | 0.727 | 0.910 | 509 | 44506 | -3.597 | **3.22E-04** | all-cause dementia |
| Five to eight years | | 0.749 | 0.677 | 0.829 | 750 | 85263 | -5.600 | **2.14E-08** | all-cause dementia |
| More than eight years | | 0.829 | 0.750 | 0.917 | 838 | 102645 | -3.656 | **2.56E-04** | all-cause dementia |
| Reference^a^ | | 1.000 | 1.000 | 1.000 | 321 | 38279 |  |  | Alzheimer’s disease |
| One year or less | | 1.151 | 0.860 | 1.540 | 53 | 6325 | 0.946 | 3.44E-01 | Alzheimer’s disease |
| Two to four years | | 0.915 | 0.771 | 1.086 | 228 | 44506 | -1.014 | 3.11E-01 | Alzheimer’s disease |
| Five to eight years | | 0.787 | 0.672 | 0.922 | 311 | 85263 | -2.974 | **2.94E-03** | Alzheimer’s disease |
| More than eight years | | 0.832 | 0.709 | 0.976 | 316 | 102645 | -2.257 | 2.40E-02 | Alzheimer’s disease |
| Reference^a^ | | 1.000 | 1.000 | 1.000 | 186 | 38279 |  |  | vascular dementia |
| One year or less | | 0.832 | 0.539 | 1.284 | 23 | 6325 | -0.830 | 4.06E-01 | vascular dementia |
| Two to four years | | 0.615 | 0.477 | 0.794 | 89 | 44506 | -3.740 | **1.84E-04** | vascular dementia |
| Five to eight years | | 0.729 | 0.589 | 0.902 | 163 | 85263 | -2.914 | **3.57E-03** | vascular dementia |
| More than eight years | | 0.746 | 0.601 | 0.925 | 169 | 102645 | -2.673 | **7.51E-03** | vascular dementia |

^a^ The reference level was never used mobile phone at least once per week.

95%CI: 95% confidence interval

^b^Significant at level P value ＜ .05/5

# Table S6. The association between the risk of dementia and length of mobile phone use (adjusted the status of APOE e4 instead of the polygenic risk scores).

|  | HR | 95%CI lower | 95%CI upper | Number of outcomes | Total | Z value | *P*^b^ | outcome |
| --- | --- | --- | --- | --- | --- | --- | --- | --- |
| Reference^a^ | 1.000 | 1.000 | 1.000 | 764 | 37023 |  |  | all-cause dementia |
| One year or less | 1.001 | 0.817 | 1.227 | 106 | 6108 | 0.009 | 9.93E-01 | all-cause dementia |
| Two to four years | 0.832 | 0.742 | 0.933 | 496 | 43055 | -3.158 | **1.59E-03** | all-cause dementia |
| Five to eight years | 0.767 | 0.692 | 0.850 | 728 | 82402 | -5.058 | **4.25E-07** | all-cause dementia |
| More than eight years | 0.852 | 0.769 | 0.943 | 819 | 99430 | -3.082 | **2.05E-03** | all-cause dementia |
| Reference^a^ | 1.000 | 1.000 | 1.000 | 304 | 37023 |  |  | Alzheimer’s disease |
| One year or less | 1.139 | 0.837 | 1.550 | 47 | 6108 | 0.831 | 4.06E-01 | Alzheimer’s disease |
| Two to four years | 0.947 | 0.795 | 1.129 | 220 | 43055 | -0.606 | 5.45E-01 | Alzheimer’s disease |
| Five to eight years | 0.820 | 0.698 | 0.964 | 301 | 82402 | -2.403 | 1.63E-02 | Alzheimer’s disease |
| More than eight years | 0.874 | 0.743 | 1.029 | 309 | 99430 | -1.615 | 1.06E-01 | Alzheimer’s disease |
| Reference^a^ | 1.000 | 1.000 | 1.000 | 177 | 37023 |  |  | vascular dementia |
| One year or less | 0.876 | 0.562 | 1.366 | 22 | 6108 | -0.582 | 5.60E-01 | vascular dementia |
| Two to four years | 0.654 | 0.506 | 0.845 | 89 | 43055 | -3.248 | **1.16E-03** | vascular dementia |
| Five to eight years | 0.757 | 0.609 | 0.941 | 158 | 82402 | -2.507 | 1.22E-02 | vascular dementia |
| More than eight years | 0.777 | 0.624 | 0.967 | 166 | 99430 | -2.257 | 2.40E-02 | vascular dementia |

^a^ The reference level was never used mobile phone at least once per week.

95%CI: 95% confidence interval

^b^Significant at level P value ＜ .05/5

|  | Table S7. The association between the risk of dementia and length of mobile phone use ( those younger than 60 years old at baseline). | | | | | | | | |
| --- | --- | --- | --- | --- | --- | --- | --- | --- | --- |
|  | | HR | 95%CI lower | 95%CI upper | Number of outcomes | Total | Z value | *P value*^a^ | outcome |
| Reference^a^ | | 1.000 | 1.000 | 1.000 | 69 | 14873 |  |  | all-cause dementia |
| One year or less | | 0.581 | 0.290 | 1.165 | 9 | 3250 | -1.529 | 1.26E-01 | all-cause dementia |
| Two to four years | | 0.730 | 0.528 | 1.010 | 80 | 26061 | -1.901 | **5.73E-02** | all-cause dementia |
| Five to eight years | | 0.470 | 0.345 | 0.641 | 100 | 53795 | -4.768 | **1.86E-06** | all-cause dementia |
| More than eight years | | 0.602 | 0.451 | 0.804 | 163 | 69322 | -3.433 | **5.97E-04** | all-cause dementia |
| Reference^a^ | | 1.000 | 1.000 | 1.000 | 25 | 14873 |  |  | Alzheimer’s disease |
| One year or less | | 0.856 | 0.327 | 2.240 | 5 | 3250 | -0.317 | 7.51E-01 | Alzheimer’s disease |
| Two to four years | | 0.766 | 0.452 | 1.299 | 32 | 26061 | -0.988 | 3.23E-01 | Alzheimer’s disease |
| Five to eight years | | 0.375 | 0.219 | 0.641 | 30 | 53795 | -3.579 | **3.44E-04** | Alzheimer’s disease |
| More than eight years | | 0.437 | 0.263 | 0.727 | 43 | 69322 | -3.189 | **1.43E-03** | Alzheimer’s disease |
| Reference^a^ | | / | / | / | 10 | 14873 |  | / | vascular dementia |
| One year or less | | / | / | / | 0 | 3250 |  | / | vascular dementia |
| Two to four years | | / | / | / | 6 | 26061 |  | / | vascular dementia |
| Five to eight years | | / | / | / | 18 | 53795 |  | / | vascular dementia |
| More than eight years | | / | / | / | 26 | 69322 |  | / | vascular dementia |

^a^ The reference level was never used mobile phone at least once per week.

95%CI: 95% confidence interval

^a^Significant at level P value ＜ 0.05/5

There were no results for the vascular dementia as the incidence of vascular dementia in participants younger than 60 was too small to analysis.

|  | Table S8. The association between the risk of dementia and length of mobile phone use ( those older than 60 years old at baseline). | | | | | | | | |
| --- | --- | --- | --- | --- | --- | --- | --- | --- | --- |
|  | | HR | 95%CI lower | 95%CI upper | Number of outcomes | Total | Z value | *P*^b^ | outcome |
| Reference^a^ | | 1.000 | 1.000 | 1.000 | 724 | 23406 |  |  | all-cause dementia |
| One year or less | | 1.031 | 0.840 | 1.264 | 106 | 3075 | 0.290 | 7.72E-01 | all-cause dementia |
| Two to four years | | 0.745 | 0.661 | 0.840 | 430 | 18446 | -4.802 | **1.57E-06** | all-cause dementia |
| Five to eight years | | 0.703 | 0.632 | 0.782 | 650 | 31468 | -6.470 | **9.79E-11** | all-cause dementia |
| More than eight years | | 0.725 | 0.651 | 0.807 | 676 | 33324 | -5.864 | **4.52E-09** | all-cause dementia |
| Reference^a^ | | 1.000 | 1.000 | 1.000 | 296 | 23406 |  |  | Alzheimer’s disease |
| One year or less | | 1.140 | 0.840 | 1.547 | 48 | 3075 | 0.839 | 4.02E-01 | Alzheimer’s disease |
| Two to four years | | 0.825 | 0.688 | 0.989 | 197 | 18446 | -2.076 | 3.80E-02 | Alzheimer’s disease |
| Five to eight years | | 0.738 | 0.626 | 0.870 | 281 | 31468 | -3.613 | **3.03E-04** | Alzheimer’s disease |
| More than eight years | | 0.745 | 0.630 | 0.881 | 274 | 33324 | -3.439 | **5.84E-04** | Alzheimer’s disease |
| Reference^a^ | | 1.000 | 1.000 | 1.000 | 176 | 23406 |  |  | vascular dementia |
| One year or less | | 0.883 | 0.571 | 1.364 | 23 | 3075 | -0.562 | 5.74E-01 | vascular dementia |
| Two to four years | | 0.585 | 0.450 | 0.760 | 83 | 18446 | -4.009 | **6.09E-05** | vascular dementia |
| Five to eight years | | 0.651 | 0.521 | 0.812 | 145 | 31468 | -3.800 | **1.45E-04** | vascular dementia |
| More than eight years | | 0.628 | 0.501 | 0.787 | 144 | 33324 | -4.039 | **5.37E-05** | vascular dementia |

^a^ The reference level was never used mobile phone at least once per week.

95%CI: 95% confidence interval

^b^Significant at level P value ＜ .05/5

# Table S9. The association between the risk of Parkinsonism and electronic device use.

| Variables | HR | 95%CI lower | 95%CI upper | Number of outcomes | Total | Z value | *P*^a^ | outcome | exposure |
| --- | --- | --- | --- | --- | --- | --- | --- | --- | --- |
| Left | 1.000 | 1.000 | 1.000 | 459 | 84079 |  |  | all-cause parkinsonism | Usual side of head for mobile phone use |
| Right | 0.979 | 0.869 | 1.102 | 680 | 139275 | -0.354 | .724 | all-cause parkinsonism | Usual side of head for mobile phone use |
| Equally left and right | 1.023 | 0.800 | 1.309 | 74 | 15119 | 0.185 | .854 | all-cause parkinsonism | Usual side of head for mobile phone use |
| less than 5 mins | 1.000 | 1.000 | 1.000 | 312 | 46861 |  |  | all-cause parkinsonism | Weekly usage of mobile phone in last 3 months |
| ≥ 5 mins | 1.044 | 0.916 | 1.191 | 901 | 191612 | 0.645 | .519 | all-cause parkinsonism | Weekly usage of mobile phone in last 3 months |
| no change | 1.000 | 1.000 | 1.000 | 706 | 124344 |  |  | all-cause parkinsonism | Difference in mobile phone use compared to two years previously |
| more frequenct | 0.951 | 0.809 | 1.119 | 188 | 31551 | -0.606 | .544 | all-cause parkinsonism | Difference in mobile phone use compared to two years previously |
| less frequent | 0.949 | 0.830 | 1.085 | 319 | 82578 | -0.765 | .444 | all-cause parkinsonism | Difference in mobile phone use compared to two years previously |
| never/almost never | 1.000 | 1.000 | 1.000 | 1039 | 191825 |  |  | all-cause parkinsonism | Hands-free device/speakerphone use with mobile phone in last 3 month |
| used | 0.928 | 0.785 | 1.098 | 174 | 46648 | -0.87 | .385 | all-cause parkinsonism | Hands-free device/speakerphone use with mobile phone in last 3 month |
| Never used mobile phone at least once per week | 1.000 | 1.000 | 1.000 | 365 | 38192 |  |  | all-cause parkinsonism | length of mobile phone use |
| One year or less | 1.038 | 0.774 | 1.393 | 51 | 6316 | 0.252 | .801 | all-cause parkinsonism | length of mobile phone use |
| Two to four years | 0.891 | 0.757 | 1.048 | 251 | 44446 | -1.394 | .163 | all-cause parkinsonism | length of mobile phone use |
| Five to eight years | 0.862 | 0.747 | 0.994 | 419 | 85160 | -2.046 | .041 | all-cause parkinsonism | length of mobile phone use |
| More than eight years | 0.873 | 0.759 | 1.006 | 492 | 102551 | -1.880 | .060 | all-cause parkinsonism | length of mobile phone use |
| Left | 1.000 | 1.000 | 1.000 | 408 | 84079 |  |  | Parkinson’s disease | Usual side of head for mobile phone use |
| Right | 0.990 | 0.874 | 1.123 | 611 | 139275 | -0.149 | .881 | Parkinson’s disease | Usual side of head for mobile phone use |
| Equally left and right | 1.034 | 0.797 | 1.342 | 66 | 15119 | 0.252 | .801 | Parkinson’s disease | Usual side of head for mobile phone use |
| less than 5 mins | 1.000 | 1.000 | 1.000 | 282 | 46861 |  |  | Parkinson’s disease | Weekly usage of mobile phone in last 3 months |
| ≥ 5 mins | 1.047 | 0.912 | 1.203 | 803 | 191612 | 0.652 | .514 | Parkinson’s disease | Weekly usage of mobile phone in last 3 months |
| no change | 1.000 | 1.000 | 1.000 | 636 | 124344 |  |  | Parkinson’s disease | Difference in mobile phone use compared to two years previously |
| more frequenct | 0.950 | 0.801 | 1.127 | 170 | 31551 | -0.585 | .558 | Parkinson’s disease | Difference in mobile phone use compared to two years previously |
| less frequent | 0.939 | 0.814 | 1.083 | 279 | 82578 | -0.861 | .389 | Parkinson’s disease | Difference in mobile phone use compared to two years previously |
| never/almost never | 1.000 | 1.000 | 1.000 | 929 | 191825 |  |  | Parkinson’s disease | Hands-free device/speakerphone use with mobile phone in last 3 month |
| used | 0.937 | 0.786 | 1.119 | 156 | 46648 | -0.716 | .474 | Parkinson’s disease | Hands-free device/speakerphone use with mobile phone in last 3 month |
| Never used mobile phone at least once per week | 1.000 | 1.000 | 1.000 | 330 | 38192 |  |  | Parkinson’s disease | length of mobile phone use |
| One year or less | 1.055 | 0.774 | 1.437 | 46 | 6316 | 0.339 | .735 | Parkinson’s disease | length of mobile phone use |
| Two to four years | 0.886 | 0.746 | 1.052 | 221 | 44446 | -1.378 | .168 | Parkinson’s disease | length of mobile phone use |
| Five to eight years | 0.868 | 0.747 | 1.009 | 375 | 85160 | -1.844 | .065 | Parkinson’s disease | length of mobile phone use |
| More than eight years | 0.877 | 0.756 | 1.017 | 443 | 102551 | -1.732 | .083 | Parkinson’s disease | length of mobile phone use |
| Never/rarely | 1.000 | 1.000 | 1.000 | 1312 | 218586 |  |  | all-cause parkinsonism | Plays computer games |
| Sometimes | 0.933 | 0.811 | 1.073 | 234 | 48861 | -0.970 | .332 | all-cause parkinsonism | Plays computer games |
| Often | 0.604 | 0.425 | 0.859 | 32 | 9218 | -2.811 | **.005** | all-cause parkinsonism | Plays computer games |
| Never/rarely | 1.000 | 1.000 | 1.000 | 1175 | 218586 |  |  | Parkinson’s disease | Plays computer games |
| Sometimes | 0.957 | 0.826 | 1.109 | 213 | 48861 | -0.583 | .560 | Parkinson’s disease | Plays computer games |
| Often | 0.574 | 0.392 | 0.841 | 27 | 9218 | -2.846 | **.004** | Parkinson’s disease | Plays computer games |

95%CI: 95% confidence interval

^a^Significant at level P value ＜ .05/5

# Table S10. The association between the risk of Parkinsonism and length of mobile phone use (excluded those who had outcomes within five years after baseline).

| Variables | HR | 95%CI lower | 95%CI upper | Number of outcomes | Total | Z value | *P*^a^ | outcome | exposure |
| --- | --- | --- | --- | --- | --- | --- | --- | --- | --- |
| Left | 1.000 | 1.000 | 1.000 | 403 | 84023 |  |  | all-cause parkinsonism | Usual side of head for mobile phone use |
| Right | 0.942 | 0.829 | 1.070 | 575 | 139170 | -0.920 | .357 | all-cause parkinsonism | Usual side of head for mobile phone use |
| Equally left and right | 0.972 | 0.743 | 1.271 | 62 | 15107 | -0.208 | .835 | all-cause parkinsonism | Usual side of head for mobile phone use |
| less than 5 mins | 1.000 | 1.000 | 1.000 | 260 | 46809 |  |  | all-cause parkinsonism | Weekly usage of mobile phone in last 3 months |
| ≥ 5 mins | 1.081 | 0.936 | 1.247 | 780 | 191491 | 1.060 | .289 | all-cause parkinsonism | Weekly usage of mobile phone in last 3 months |
| no change | 1.000 | 1.000 | 1.000 | 618 | 124256 |  |  | all-cause parkinsonism | Difference in mobile phone use compared to two years previously |
| more frequenct | 0.927 | 0.778 | 1.105 | 160 | 31523 | -0.845 | .398 | all-cause parkinsonism | Difference in mobile phone use compared to two years previously |
| less frequent | 0.888 | 0.767 | 1.028 | 262 | 82521 | -1.587 | .113 | all-cause parkinsonism | Difference in mobile phone use compared to two years previously |
| never/almost never | 1.000 | 1.000 | 1.000 | 889 | 191675 |  |  | all-cause parkinsonism | Hands-free device/speakerphone use with mobile phone in last 3 month |
| used | 0.930 | 0.776 | 1.113 | 151 | 46625 | -0.796 | .426 | all-cause parkinsonism | Hands-free device/speakerphone use with mobile phone in last 3 month |
| Never used mobile phone at least once per week | 1.000 | 1.000 | 1.000 | 310 | 38137 |  |  | all-cause parkinsonism | length of mobile phone use |
| One year or less | 1.016 | 0.736 | 1.403 | 42 | 6307 | 0.097 | .922 | all-cause parkinsonism | length of mobile phone use |
| Two to four years | 0.872 | 0.730 | 1.041 | 208 | 44403 | -1.519 | .129 | all-cause parkinsonism | length of mobile phone use |
| Five to eight years | 0.876 | 0.751 | 1.023 | 363 | 85104 | -1.677 | .094 | all-cause parkinsonism | length of mobile phone use |
| More than eight years | 0.883 | 0.758 | 1.028 | 427 | 102486 | -1.601 | .109 | all-cause parkinsonism | length of mobile phone use |
| Left | 1.000 | 1.000 | 1.000 | 359 | 84023 |  |  | Parkinson’s disease | Usual side of head for mobile phone use |
| Right | 0.957 | 0.837 | 1.095 | 520 | 139170 | -0.639 | .523 | Parkinson’s disease | Usual side of head for mobile phone use |
| Equally left and right | 0.991 | 0.748 | 1.315 | 56 | 15107 | -0.060 | .952 | Parkinson’s disease | Usual side of head for mobile phone use |
| less than 5 mins | 1.000 | 1.000 | 1.000 | 235 | 46809 |  |  | Parkinson’s disease | Weekly usage of mobile phone in last 3 months |
| ≥ 5 mins | 1.088 | 0.936 | 1.266 | 700 | 191491 | 1.102 | .271 | Parkinson’s disease | Weekly usage of mobile phone in last 3 months |
| no change | 1.000 | 1.000 | 1.000 | 560 | 124256 |  |  | Parkinson’s disease | Difference in mobile phone use compared to two years previously |
| more frequenct | 0.921 | 0.766 | 1.107 | 145 | 31523 | -0.877 | .380 | Parkinson’s disease | Difference in mobile phone use compared to two years previously |
| less frequent | 0.875 | 0.749 | 1.022 | 230 | 82521 | -1.682 | .093 | Parkinson’s disease | Difference in mobile phone use compared to two years previously |
| never/almost never | 1.000 | 1.000 | 1.000 | 797 | 191675 |  |  | Parkinson’s disease | Hands-free device/speakerphone use with mobile phone in last 3 month |
| used | 0.950 | 0.786 | 1.147 | 138 | 46625 | -0.537 | .591 | Parkinson’s disease | Hands-free device/speakerphone use with mobile phone in last 3 month |
| Never used mobile phone at least once per week | 1.000 | 1.000 | 1.000 | 282 | 38137 |  |  | Parkinson’s disease | length of mobile phone use |
| One year or less | 1.056 | 0.755 | 1.477 | 39 | 6307 | 0.319 | .750 | Parkinson’s disease | length of mobile phone use |
| Two to four years | 0.870 | 0.722 | 1.049 | 185 | 44403 | -1.458 | .145 | Parkinson’s disease | length of mobile phone use |
| Five to eight years | 0.879 | 0.748 | 1.034 | 326 | 85104 | -1.559 | .119 | Parkinson’s disease | length of mobile phone use |
| More than eight years | 0.879 | 0.749 | 1.031 | 385 | 102486 | -1.586 | .113 | Parkinson’s disease | length of mobile phone use |
| Never/rarely | 1.000 | 1.000 | 1.000 | 1117 | 218391 |  |  | all-cause parkinsonism | Plays computer games |
| Sometimes | 0.948 | 0.815 | 1.102 | 203 | 48830 | -0.695 | .487 | all-cause parkinsonism | Plays computer games |
| Often | 0.664 | 0.462 | 0.956 | 30 | 9216 | -2.204 | .028 | all-cause parkinsonism | Plays computer games |
| Never/rarely | 1.000 | 1.000 | 1.000 | 1006 | 218391 |  |  | Parkinson’s disease | Plays computer games |
| Sometimes | 0.973 | 0.831 | 1.139 | 186 | 48830 | -0.340 | .734 | Parkinson’s disease | Plays computer games |
| Often | 0.621 | 0.417 | 0.924 | 25 | 9216 | -2.351 | .019 | Parkinson’s disease | Plays computer games |

95%CI: 95% confidence interval

^a^Significant at level P value ＜ .05/5

# Table S11. The association between the risk of Parkinsonism and length of mobile phone use (excluded participants whose source of outcomes were not from the hospital or the death registration).

| Variables | HR | 95%CI lower | 95%CI upper | Number of outcomes | Total | Z value | *P*^a^ | outcome | exposure |
| --- | --- | --- | --- | --- | --- | --- | --- | --- | --- |
| Left | 1.000 | 1.000 | 1.000 | 456 | 84076 |  |  | all-cause parkinsonism | Usual side of head for mobile phone use |
| Right | 0.983 | 0.873 | 1.107 | 678 | 139273 | -0.284 | .776 | all-cause parkinsonism | Usual side of head for mobile phone use |
| Equally left and right | 1.031 | 0.806 | 1.319 | 74 | 15119 | 0.243 | .808 | all-cause parkinsonism | Usual side of head for mobile phone use |
| less than 5 mins | 1.000 | 1.000 | 1.000 | 311 | 46860 |  |  | all-cause parkinsonism | Weekly usage of mobile phone in last 3 months |
| ≥ 5 mins | 1.045 | 0.916 | 1.192 | 897 | 191608 | 0.657 | .511 | all-cause parkinsonism | Weekly usage of mobile phone in last 3 months |
| no change | 1.000 | 1.000 | 1.000 | 704 | 124342 |  |  | all-cause parkinsonism | Difference in mobile phone use compared to two years previously |
| more frequenct | 0.944 | 0.802 | 1.111 | 186 | 31549 | -0.693 | .488 | all-cause parkinsonism | Difference in mobile phone use compared to two years previously |
| less frequent | 0.950 | 0.831 | 1.087 | 318 | 82577 | -0.747 | .455 | all-cause parkinsonism | Difference in mobile phone use compared to two years previously |
| never/almost never | 1.000 | 1.000 | 1.000 | 1035 | 191821 |  |  | all-cause parkinsonism | Hands-free device/speakerphone use with mobile phone in last 3 month |
| used | 0.931 | 0.787 | 1.101 | 173 | 46647 | -0.833 | .405 | all-cause parkinsonism | Hands-free device/speakerphone use with mobile phone in last 3 month |
| Never used mobile phone at least once per week | 1.000 | 1.000 | 1.000 | 361 | 38188 |  |  | all-cause parkinsonism | length of mobile phone use |
| One year or less | 1.050 | 0.783 | 1.408 | 51 | 6316 | 0.325 | .746 | all-cause parkinsonism | length of mobile phone use |
| Two to four years | 0.897 | 0.762 | 1.056 | 250 | 44445 | -1.303 | .192 | all-cause parkinsonism | length of mobile phone use |
| Five to eight years | 0.866 | 0.750 | 0.999 | 416 | 85157 | -1.969 | .049 | all-cause parkinsonism | length of mobile phone use |
| More than eight years | 0.885 | 0.768 | 1.019 | 491 | 102550 | -1.698 | .089 | all-cause parkinsonism | length of mobile phone use |
| Left | 1.000 | 1.000 | 1.000 | 405 | 84076 |  |  | Parkinson’s disease | Usual side of head for mobile phone use |
| Right | 0.995 | 0.878 | 1.129 | 609 | 139273 | -0.076 | .939 | Parkinson’s disease | Usual side of head for mobile phone use |
| Equally left and right | 1.043 | 0.803 | 1.353 | 66 | 15119 | 0.315 | .753 | Parkinson’s disease | Usual side of head for mobile phone use |
| less than 5 mins | 1.000 | 1.000 | 1.000 | 281 | 46860 |  |  | Parkinson’s disease | Weekly usage of mobile phone in last 3 months |
| ≥ 5 mins | 1.048 | 0.912 | 1.204 | 799 | 191608 | 0.665 | .506 | Parkinson’s disease | Weekly usage of mobile phone in last 3 months |
| no change | 1.000 | 1.000 | 1.000 | 634 | 124342 |  |  | Parkinson’s disease | Difference in mobile phone use compared to two years previously |
| more frequenct | 0.943 | 0.794 | 1.119 | 168 | 31549 | -0.676 | .499 | Parkinson’s disease | Difference in mobile phone use compared to two years previously |
| less frequent | 0.940 | 0.815 | 1.085 | 278 | 82577 | -0.842 | .400 | Parkinson’s disease | Difference in mobile phone use compared to two years previously |
| never/almost never | 1.000 | 1.000 | 1.000 | 925 | 191821 |  |  | Parkinson’s disease | Hands-free device/speakerphone use with mobile phone in last 3 month |
| used | 0.941 | 0.788 | 1.123 | 155 | 46647 | -0.676 | .499 | Parkinson’s disease | Hands-free device/speakerphone use with mobile phone in last 3 month |
| Never used mobile phone at least once per week | 1.000 | 1.000 | 1.000 | 326 | 38188 |  |  | Parkinson’s disease | length of mobile phone use |
| One year or less | 1.068 | 0.784 | 1.455 | 46 | 6316 | 0.416 | .678 | Parkinson’s disease | length of mobile phone use |
| Two to four years | 0.893 | 0.752 | 1.061 | 220 | 44445 | -1.283 | .200 | Parkinson’s disease | length of mobile phone use |
| Five to eight years | 0.873 | 0.750 | 1.015 | 372 | 85157 | -1.762 | .078 | Parkinson’s disease | length of mobile phone use |
| More than eight years | 0.890 | 0.767 | 1.032 | 442 | 102550 | -1.540 | .124 | Parkinson’s disease | length of mobile phone use |
| Never/rarely | 1.000 | 1.000 | 1.000 | 1303 | 218577 |  | 1.000 | all-cause parkinsonism | Plays computer games |
| Sometimes | 0.939 | 0.816 | 1.080 | 234 | 48861 | -0.880 | .379 | all-cause parkinsonism | Plays computer games |
| Often | 0.608 | 0.428 | 0.864 | 32 | 9218 | -2.777 | **.005** | all-cause parkinsonism | Plays computer games |
| Never/rarely | 1.000 | 1.000 | 1.000 | 1166 | 218577 |  | 1.000 | Parkinson’s disease | Plays computer games |
| Sometimes | 0.964 | 0.832 | 1.117 | 213 | 48861 | -0.488 | .625 | Parkinson’s disease | Plays computer games |
| Often | 0.578 | 0.394 | 0.847 | 27 | 9218 | -2.811 | **.005** | Parkinson’s disease | Plays computer games |

95%CI: 95% confidence interval

^a^Significant at level P value ＜ .05/5

# Table S12. The association between the risk of Parkinsonism and electronic devices use ( those younger than 60 years old at baseline).

| Variables | HR | 95%CI lower | 95%CI upper | Number of outcomes | Total | Z value | *P*^a^ | outcome | exposure |
| --- | --- | --- | --- | --- | --- | --- | --- | --- | --- |
| Left | 1.000 | 1.000 | 1.000 | 121 | 51169 |  |  | all-cause parkinsonism | Usual side of head for mobile phone use |
| Right | 0.879 | 0.699 | 1.106 | 186 | 90814 | -1.100 | .271 | all-cause parkinsonism | Usual side of head for mobile phone use |
| Equally left and right | 1.033 | 0.676 | 1.580 | 26 | 10363 | 0.150 | .880 | all-cause parkinsonism | Usual side of head for mobile phone use |
| less than 5 mins | 1.000 | 1.000 | 1.000 | 59 | 22618 |  |  | all-cause parkinsonism | Weekly usage of mobile phone in last 3 months |
| ≥ 5 mins | 0.821 | 0.618 | 1.091 | 274 | 129728 | -1.359 | .174 | all-cause parkinsonism | Weekly usage of mobile phone in last 3 months |
| no change | 1.000 | 1.000 | 1.000 | 171 | 73858 |  |  | all-cause parkinsonism | Difference in mobile phone use compared to two years previously |
| more frequenct | 0.947 | 0.679 | 1.321 | 44 | 18325 | -0.320 | .749 | all-cause parkinsonism | Difference in mobile phone use compared to two years previously |
| less frequent | 0.888 | 0.701 | 1.125 | 118 | 60163 | -0.986 | .324 | all-cause parkinsonism | Difference in mobile phone use compared to two years previously |
| Never/rarely | 1.000 | 1.000 | 1.000 | 305 | 129291 |  |  | all-cause parkinsonism | Plays computer games |
| Sometimes | 0.787 | 0.599 | 1.035 | 63 | 32282 | -1.713 | .087 | all-cause parkinsonism | Plays computer games |
| Often | 0.393 | 0.175 | 0.884 | 6 | 5630 | -2.257 | .024 | all-cause parkinsonism | Plays computer games |
| never/almost never | 1.000 | 1.000 | 1.000 | 263 | 116190 |  |  | all-cause parkinsonism | Hands-free device/speakerphone use with mobile phone in last 3 month |
| used | 0.787 | 0.599 | 1.034 | 70 | 36156 | -1.719 | .086 | all-cause parkinsonism | Hands-free device/speakerphone use with mobile phone in last 3 month |
| Never used mobile phone at least once per week | 1.000 | 1.000 | 1.000 | 41 | 14857 |  |  | all-cause parkinsonism | length of mobile phone use |
| One year or less | 1.650 | 0.899 | 3.029 | 14 | 3246 | 1.615 | .106 | all-cause parkinsonism | length of mobile phone use |
| Two to four years | 0.891 | 0.593 | 1.339 | 55 | 26045 | -0.555 | .579 | all-cause parkinsonism | length of mobile phone use |
| Five to eight years | 0.957 | 0.669 | 1.368 | 122 | 53766 | -0.241 | .810 | all-cause parkinsonism | length of mobile phone use |
| More than eight years | 0.814 | 0.570 | 1.163 | 142 | 69289 | -1.130 | .259 | all-cause parkinsonism | length of mobile phone use |
| Left | 1.000 | 1.000 | 1.000 | 94 | 51169 |  |  | Parkinson’s disease | Usual side of head for mobile phone use |
| Right | 0.943 | 0.730 | 1.218 | 156 | 90814 | -0.450 | .652 | Parkinson’s disease | Usual side of head for mobile phone use |
| Equally left and right | 1.072 | 0.667 | 1.722 | 21 | 10363 | 0.287 | .774 | Parkinson’s disease | Usual side of head for mobile phone use |
| less than 5 mins | 1.000 | 1.000 | 1.000 | 47 | 22618 |  |  | Parkinson’s disease | Weekly usage of mobile phone in last 3 months |
| ≥ 5 mins | 0.825 | 0.600 | 1.133 | 224 | 129728 | -1.189 | .234 | Parkinson’s disease | Weekly usage of mobile phone in last 3 months |
| no change | 1.000 | 1.000 | 1.000 | 142 | 73858 |  |  | Parkinson’s disease | Difference in mobile phone use compared to two years previously |
| more frequenct | 0.862 | 0.589 | 1.262 | 33 | 18325 | -0.763 | .446 | Parkinson’s disease | Difference in mobile phone use compared to two years previously |
| less frequent | 0.867 | 0.668 | 1.125 | 96 | 60163 | -1.075 | .282 | Parkinson’s disease | Difference in mobile phone use compared to two years previously |
| Never/rarely | 1.000 | 1.000 | 1.000 | 246 | 129291 |  |  | Parkinson’s disease | Plays computer games |
| Sometimes | 0.872 | 0.651 | 1.168 | 56 | 32282 | -0.918 | .359 | Parkinson’s disease | Plays computer games |
| Often | 0.333 | 0.124 | 0.896 | 4 | 5630 | -2.178 | .029 | Parkinson’s disease | Plays computer games |
| never/almost never | 1.000 | 1.000 | 1.000 | 212 | 116190 |  |  | Parkinson’s disease | Hands-free device/speakerphone use with mobile phone in last 3 month |
| used | 0.799 | 0.592 | 1.077 | 59 | 36156 | -1.473 | .141 | Parkinson’s disease | Hands-free device/speakerphone use with mobile phone in last 3 month |
| Never used mobile phone at least once per week | 1.000 | 1.000 | 1.000 | 35 | 14857 |  |  | Parkinson’s disease | length of mobile phone use |
| One year or less | 1.530 | 0.776 | 3.016 | 11 | 3246 | 1.229 | .219 | Parkinson’s disease | length of mobile phone use |
| Two to four years | 0.808 | 0.516 | 1.266 | 43 | 26045 | -0.931 | .352 | Parkinson’s disease | length of mobile phone use |
| Five to eight years | 0.887 | 0.601 | 1.310 | 99 | 53766 | -0.601 | .548 | Parkinson’s disease | length of mobile phone use |
| More than eight years | 0.761 | 0.517 | 1.122 | 118 | 69289 | -1.379 | .168 | Parkinson’s disease | length of mobile phone use |

95%CI: 95% confidence interval

^a^Significant at level P value ＜ .05/5

# Table S13. The association between the risk of Parkinsonism and electronic devices use ( those older than 60 years old at baseline).

| Variables | HR | 95%CI lower | 95%CI upper | Number of outcomes | Total | Z value | *P*^a^ | outcome | exposure |
| --- | --- | --- | --- | --- | --- | --- | --- | --- | --- |
| Left | 1.000 | 1.000 | 1.000 | 338 | 32910 |  |  | all-cause parkinsonism | Usual side of head for mobile phone use |
| Right | 0.996 | 0.868 | 1.144 | 494 | 48461 | -0.050 | .960 | all-cause parkinsonism | Usual side of head for mobile phone use |
| Equally left and right | 0.965 | 0.713 | 1.307 | 48 | 4756 | -0.227 | .820 | all-cause parkinsonism | Usual side of head for mobile phone use |
| less than 5 mins | 1.000 | 1.000 | 1.000 | 253 | 24243 |  |  | all-cause parkinsonism | Weekly usage of mobile phone in last 3 months |
| ≥5 mins | 1.018 | 0.878 | 1.180 | 627 | 61884 | 0.236 | .813 | all-cause parkinsonism | Weekly usage of mobile phone in last 3 months |
| no change | 1.000 | 1.000 | 1.000 | 535 | 50486 |  |  | all-cause parkinsonism | Difference in mobile phone use compared to two years previously |
| more frequenct | 0.905 | 0.751 | 1.090 | 144 | 13226 | -1.055 | .291 | all-cause parkinsonism | Difference in mobile phone use compared to two years previously |
| less frequent | 0.903 | 0.767 | 1.063 | 201 | 22415 | -1.226 | .220 | all-cause parkinsonism | Difference in mobile phone use compared to two years previously |
| Never/rarely | 1.000 | 1.000 | 1.000 | 1007 | 89295 |  |  | all-cause parkinsonism | Plays computer games |
| Sometimes | 0.925 | 0.786 | 1.089 | 171 | 16579 | -0.940 | .347 | all-cause parkinsonism | Plays computer games |
| Often | 0.661 | 0.447 | 0.977 | 26 | 3588 | -2.079 | .038 | all-cause parkinsonism | Plays computer games |
| never/almost never | 1.000 | 1.000 | 1.000 | 776 | 75635 |  |  | all-cause parkinsonism | Hands-free device/speakerphone use with mobile phone in last 3 month |
| used | 0.876 | 0.710 | 1.081 | 104 | 10492 | -1.232 | .218 | all-cause parkinsonism | Hands-free device/speakerphone use with mobile phone in last 3 month |
| Never used mobile phone at least once per week | 1.000 | 1.000 | 1.000 | 324 | 23335 |  |  | all-cause parkinsonism | length of mobile phone use |
| One year or less | 0.887 | 0.631 | 1.247 | 37 | 3070 | -0.692 | .489 | all-cause parkinsonism | length of mobile phone use |
| Two to four years | 0.832 | 0.696 | 0.995 | 196 | 18401 | -2.019 | .043 | all-cause parkinsonism | length of mobile phone use |
| Five to eight years | 0.747 | 0.637 | 0.875 | 297 | 31394 | -3.601 | **＜.001** | all-cause parkinsonism | length of mobile phone use |
| More than eight years | 0.774 | 0.663 | 0.904 | 350 | 33262 | -3.229 | **.001** | all-cause parkinsonism | length of mobile phone use |
| Left | 1.000 | 1.000 | 1.000 | 314 | 32910 |  |  | Parkinson’s disease | Usual side of head for mobile phone use |
| Right | 0.986 | 0.854 | 1.139 | 455 | 48461 | -0.189 | .850 | Parkinson’s disease | Usual side of head for mobile phone use |
| Equally left and right | 0.972 | 0.711 | 1.330 | 45 | 4756 | -0.176 | .861 | Parkinson’s disease | Usual side of head for mobile phone use |
| less than 5 mins | 1.000 | 1.000 | 1.000 | 235 | 24243 |  |  | Parkinson’s disease | Weekly usage of mobile phone in last 3 months |
| ≥ 5 mins | 1.008 | 0.865 | 1.175 | 579 | 61884 | 0.104 | .917 | Parkinson’s disease | Weekly usage of mobile phone in last 3 months |
| no change | 1.000 | 1.000 | 1.000 | 494 | 50486 |  |  | Parkinson’s disease | Difference in mobile phone use compared to two years previously |
| more frequenct | 0.925 | 0.764 | 1.120 | 137 | 13226 | -0.798 | .425 | Parkinson’s disease | Difference in mobile phone use compared to two years previously |
| less frequent | 0.891 | 0.751 | 1.057 | 183 | 22415 | -1.324 | .186 | Parkinson’s disease | Difference in mobile phone use compared to two years previously |
| Never/rarely | 1.000 | 1.000 | 1.000 | 929 | 89295 |  |  | Parkinson’s disease | Plays computer games |
| Sometimes | 0.919 | 0.775 | 1.089 | 157 | 16579 | -0.977 | .329 | Parkinson’s disease | Plays computer games |
| Often | 0.635 | 0.419 | 0.961 | 23 | 3588 | -2.150 | .032 | Parkinson’s disease | Plays computer games |
| never/almost never | 1.000 | 1.000 | 1.000 | 717 | 75635 |  |  | Parkinson’s disease | Hands-free device/speakerphone use with mobile phone in last 3 month |
| used | 0.870 | 0.699 | 1.082 | 97 | 10492 | -1.253 | .210 | Parkinson’s disease | Hands-free device/speakerphone use with mobile phone in last 3 month |
| Never used mobile phone at least once per week | 1.000 | 1.000 | 1.000 | 295 | 23335 |  |  | Parkinson’s disease | length of mobile phone use |
| One year or less | 0.928 | 0.653 | 1.318 | 35 | 3070 | -0.420 | .674 | Parkinson’s disease | length of mobile phone use |
| Two to four years | 0.833 | 0.691 | 1.005 | 178 | 18401 | -1.909 | .056 | Parkinson’s disease | length of mobile phone use |
| Five to eight years | 0.760 | 0.644 | 0.897 | 276 | 31394 | -3.251 | **.001** | Parkinson’s disease | length of mobile phone use |
| More than eight years | 0.777 | 0.660 | 0.913 | 325 | 33262 | -3.059 | **.002** | Parkinson’s disease | length of mobile phone use |

95%CI: 95% confidence interval

^a^Significant at level P value ＜ .05/5

# Table S14. Demographic characteristics of participants included in the neuroimage analysis.

| **Variable** | **N = 35643** |
| --- | --- |
| Usual side of head for mobile phone use |  |
| Left | 10913 (35%) |
| Right | 18046 (58%) |
| Equally left and right | 2139 (7%) |
| Do not need to answer | 4,545 |
| Plays computer games |  |
| Never/rarely | 28017 (79%) |
| Sometimes | 6458 (18%) |
| Often | 1168 (3%) |
| Weekly usage of mobile phone in last 3 months |  |
| less than 5 mins | 6170 (20%) |
| ≥ 5 mins | 24928 (80%) |
| Do not need to answer | 4,545 |
| Hands-free device/speakerphone use with mobile phone in last 3 month | |
| never/almost never | 24607 (79%) |
| used | 6491 (21%) |
| Do not need to answer | 4,545 |
| Difference in mobile phone use compared to two years previously | |
| no change | 15846 (51%) |
| more frequenct | 4042 (13%) |
| less frequent | 11210 (36%) |
| Do not need to answer | 4,545 |
| Length of mobile phone use |  |
| Never used mobile phone at least once per week | 4545 (13%) |
| One year or less | 628 (2%) |
| Two to four years | 5370 (15%) |
| Five to eight years | 11373 (32%) |
| More than eight years | 13727 (39%) |
| **age** | 54.77±7.51 |
| **sex** |  |
| female | 18459 (52%) |
| male | 17184 (48%) |
| **BMI** | 26.52±4.19 |
| **smoke** |  |
| Yes | 21671 (61%) |
| No | 13972 (39%) |
| **drink** |  |
| Yes | 806 (2%) |
| No | 34837 (98%) |
| **hypertension** |  |
| Yes | 7324 (21%) |
| No | 28319 (79%) |
| **all diabetes status** |  |
| Yes | 34689 (97%) |
| No | 954 (3%) |
| **education** |  |
| college/university degree | 18320 (51%) |
| Lower degree | 17323 (49%) |
| Townsend deprivation index | -1.88±2.73 |
| Average Total Household Income Before Tax |  |
| 18000 To 30999 | 4055 (11%) |
| 31000 To 51999 | 7747 (22%) |
| 52000 To 100000 | 10821 (30%) |
| Do Not Know | 10193 (29%) |
| Greater Than 100000 | 2827 (8%) |
| white matter hyperintensities volume | 4983.57±6488.76 |
| whole white matter volume | 701050.34±40676.80 |
| whole gray matter volume | 791647.08±48258.25 |
| **Handness** |  |
| Right | 31721 (89%) |
| Left | 3922 (11%) |

# Table S15. Demographic characteristics of participants included in the sensitivity analysis of neuroimage analysis.

| **Variable** | **N = 26870** |
| --- | --- |
| Usual side of head for mobile phone use |  |
| Left | 8259 (35%) |
| Right | 13715 (58%) |
| Equally left and right | 1598 (7%) |
| Do not need to answer | 3,298 |
| Plays computer games |  |
| Never/rarely | 21170 (79%) |
| Sometimes | 4848 (18%) |
| Often | 852 (3%) |
| Weekly usage of mobile phone in last 3 months | |
| less than 5 mins | 4568 (19%) |
| ≥ 5 mins | 19004 (81%) |
| Do not need to answer | 3,298 |
| Hands-free device/speakerphone use with mobile phone in last 3 month | |
| never/almost never | 18437 (78%) |
| used | 5135 (22%) |
| Do not need to answer | 3,298 |
| Difference in mobile phone use compared to two years previously | |
| no change | 11910 (51%) |
| more frequenct | 3166 (13%) |
| less frequent | 8496 (36%) |
| Do not need to answer | 3,298 |
| Length of mobile phone use |  |
| Never used mobile phone at least once per week | 3298 (12%) |
| One year or less | 449 (2%) |
| Two to four years | 3889 (14%) |
| Five to eight years | 8473 (32%) |
| More than eight years | 10761 (40%) |
| **age** | 54.68±7.50 |
| **sex** |  |
| female | 13393 (50%) |
| male | 13477 (50%) |
| **BMI** | 26.54±4.15 |
| **smoke** |  |
| Yes | 16364 (61%) |
| No | 10506 (39%) |
| **drink** |  |
| Yes | 575 (2%) |
| No | 26295 (98%) |
| **hypertension** |  |
| Yes | 5507 (20%) |
| No | 21363 (80%) |
| **all diabetes status** |  |
| Yes | 26143 (97%) |
| No | 727 (3%) |
| **education** |  |
| college/university degree | 13426 (50%) |
| Lower degree | 13444 (50%) |
| Townsend deprivation index | -1.92±2.70 |
| Average Total Household Income Before Tax |  |
| 18000 To 30999 | 2837 (11%) |
| 31000 To 51999 | 5551 (21%) |
| 52000 To 100000 | 8145 (30%) |
| Do Not Know | 7981 (30%) |
| Greater Than 100000 | 2356 (9%) |
| white matter hyperintensities volume | 4930.31±6329.75 |
| whole white matter volume | 701419.26±40557.33 |
| whole gray matter volume | 791372.18±48164.81 |
| **Handness** |  |
| Right | 23925 (89%) |
| Left | 2945 (11%) |
| **emotional score** | 4.07±3.16 |
| **regulart PA** |  |
| not meeting the criteria | 3604 (13%) |
| meeting the criteria | 23266 (87%) |

# Table S16. Sensitivity analysis of areas where gray matter metrics related to the use of electronic devices.

| **Areas** | **β** | **Standard Error** | ***P* value** | **T value** |
| --- | --- | --- | --- | --- |
| **Length of mobile phone use (Reference: Never used mobile phone at least once per week)** | | | |  |
| **More than eight years** |  |  |  |  |
| Mean thickness of G-precuneus (left) | 0.013 | 0.004 | 1.73E-04 | 3.756 |
| Mean thickness of S-subparietal (left) | 0.013 | 0.003 | 4.69E-05 | 4.071 |
| Mean thickness of G-cingul-Post-dorsal (right) | 0.015 | 0.004 | 1.19E-04 | 3.848 |
| Mean thickness of G-orbital (right) | 0.013 | 0.004 | 1.83E-04 | 3.742 |
| Mean thickness of G-pariet-inf-Supramar (right) | 0.013 | 0.003 | 1.28E-04 | 3.831 |
| Mean thickness of G-precuneus (right) | 0.014 | 0.004 | 5.29E-05 | 4.043 |
| Mean thickness of G-temporal-middle (right) | 0.013 | 0.003 | 8.19E-05 | 3.940 |
| Mean thickness of S-subparietal (right) | 0.014 | 0.003 | 1.09E-05 | 4.399 |
| Mean thickness of S-temporal-sup (right) | 0.010 | 0.003 | 1.62E-04 | 3.772 |
| Mean thickness of G-orbital (left) | 0.013 | 0.003 | 8.77E-05 | 3.923 |
| **Two to four years** |  |  |  |  |
| Mean thickness of G-orbital (left) | 0.014 | 0.004 | 2.46E-04 | 3.667 |
| **Weekly usage of mobile phone in last 3 months (Reference: < 5 minutes)** | | |  |  |
| Volume of S-collat-transv-post (right) | -10.344 | 2.801 | 2.22E-04 | -3.693 |
| **Hands-free device/speakerphone use with mobile phone in last 3 months (Reference:never/almost never)** | | | |  |
| **Used** |  |  |  |  |
| Area of G-parietal-sup (right) | -14.653 | 4.068 | 3.16E-04 | -3.602 |
| **Usual side of head for mobile phone use (Reference: Left)** |  |  |  |  |
| **Right** |  |  |  |  |
| Area of S-temporal-sup (right) | 24.515 | 6.666 | 2.36E-04 | 3.678 |
| Mean thickness of S-front-middle (right) | -0.007 | 0.002 | 1.63E-04 | -3.771 |
| Area of G+S-cingul-Mid-Ant (left) | 8.076 | 1.951 | 3.51E-05 | 4.139 |
| Area of G-cingul-Post-dorsal (left) | 4.540 | 1.018 | 8.18E-06 | 4.461 |
| Area of G-cingul-Post-ventral (left) | 2.137 | 0.533 | 6.15E-05 | 4.008 |
| **Plays computer games (Reference: Never/rarely)** |  |  |  |  |
| **Often** |  |  |  |  |
| Volume of G+S-cingul-Ant (left) | 80.891 | 22.305 | 2.88E-04 | 3.627 |
| Volume of G-front-inf-Triangul (left) | 67.087 | 17.679 | 1.48E-04 | 3.795 |
| **Sometimes** |  |  |  |  |
| Volume of G+S-subcentral (left) | -28.769 | 7.308 | 8.29E-05 | -3.937 |
| Volume of G-oc-temp-med-Parahip (left) | -43.575 | 11.256 | 1.09E-04 | -3.871 |
| Volume of S-postcentral (left) | -53.568 | 11.534 | 3.43E-06 | -4.644 |
| Area of S-intrapariet+P-trans (right) | -19.343 | 5.369 | 3.15E-04 | -3.603 |
| Area of S-postcentral (right) | -16.805 | 4.353 | 1.13E-04 | -3.861 |
| Area of S-subparietal (right) | -10.244 | 2.637 | 1.03E-04 | -3.884 |
| Volume of G-oc-temp-med-Parahip (right) | -37.590 | 10.171 | 2.20E-04 | -3.696 |
| Volume of S-intrapariet+P-trans (right) | -48.411 | 12.570 | 1.18E-04 | -3.851 |
| Volume of S-postcentral (right) | -40.383 | 10.333 | 9.32E-05 | -3.908 |
| Area of S-postcentral (left) | -21.382 | 4.788 | 8.03E-06 | -4.465 |

# Table S17. Sensitivity analysis of areas where DTI metrics related to the use of electronic devices.

| **Areas** | **β** | **Standard Error** | ***P* value** | **T value** |
| --- | --- | --- | --- | --- |
| **Length of mobile phone use (Reference: Never used mobile phone at least once per week)** | | | |  |
| **More than eight years** |  |  |  |  |
| Mean FA in pontine crossing tract on FA skeleton | 0.003 | 0.001 | 6.42E-05 | 3.998 |
| Mean MO in posterior thalamic radiation on FA skeleton (right) | 0.006 | 0.001 | 5.90E-05 | 4.017 |
| Mean MO in external capsule on FA skeleton (right) | 0.005 | 0.001 | 4.53E-05 | 4.080 |
| Mean MO in external capsule on FA skeleton (left) | 0.006 | 0.001 | 7.20E-07 | 4.957 |
| Mean OD in posterior thalamic radiation on FA skeleton (right) | -0.001 | 0.000 | 1.45E-04 | -3.800 |
| **Two to four years** |  |  |  |  |
| Mean MO in posterior thalamic radiation on FA skeleton (right) | 0.007 | 0.002 | 5.82E-05 | 4.021 |
| Mean OD in fornix on FA skeleton | -0.014 | 0.003 | 2.02E-05 | -4.264 |
| **Weekly usage of mobile phone in last 3 months (Reference: < 5 minutes)** | |  |  |  |
| Mean OD in cerebral peduncle on FA skeleton (left) | 0.001 | 0.000 | 7.44E-05 | 3.963 |
| **Usual side of head for mobile phone use (Reference: Left)** |  |  |  |  |
| **Right** |  |  |  |  |
| Mean FA in posterior limb of internal capsule on FA skeleton (right) | 0.001 | 0.000 | 1.77E-04 | 3.750 |
| **Plays computer games (Reference: Never/rarely)** |  |  |  |  |
| **Sometimes** |  |  |  |  |
| Mean MO in splenium of corpus callosum on FA skeleton | -0.002 | 0.000 | 1.40E-04 | -3.808 |
| Mean OD in splenium of corpus callosum on FA skeleton | 0.001 | 0.000 | 3.30E-05 | 4.152 |
